# Systematic chemical-genetic and chemical-chemical interaction datasets for prediction of compound synergism

**DOI:** 10.1038/sdata.2016.95

**Published:** 2016-11-22

**Authors:** Jan Wildenhain, Michaela Spitzer, Sonam Dolma, Nick Jarvik, Rachel White, Marcia Roy, Emma Griffiths, David S. Bellows, Gerard D. Wright, Mike Tyers

**Affiliations:** 1 Wellcome Trust Centre for Cell Biology, University of Edinburgh, Edinburgh EH9 3JR, UK; 2 Michael G. DeGroote Institute for Infectious Disease Research, McMaster University, Hamilton, Ontario, Canada L8N 3Z5; 3 Lunenfeld-Tanenbaum Research Institute, Mount Sinai Hospital, Toronto, Ontario, Canada M5G 1X5; 4 Institute for Research in Immunology and Cancer, Université de Montréal, Montréal, Quebec, Canada H3C 3J7

**Keywords:** High-throughput screening, Chemical genetics, Networks and systems biology, Small molecules, Screening

## Abstract

The network structure of biological systems suggests that effective therapeutic intervention may require combinations of agents that act synergistically. However, a dearth of systematic chemical combination datasets have limited the development of predictive algorithms for chemical synergism. Here, we report two large datasets of linked chemical-genetic and chemical-chemical interactions in the budding yeast *Saccharomyces cerevisiae*. We screened 5,518 unique compounds against 242 diverse yeast gene deletion strains to generate an extended chemical-genetic matrix (CGM) of 492,126 chemical-gene interaction measurements. This CGM dataset contained 1,434 genotype-specific inhibitors, termed cryptagens. We selected 128 structurally diverse cryptagens and tested all pairwise combinations to generate a benchmark dataset of 8,128 pairwise chemical-chemical interaction tests for synergy prediction, termed the cryptagen matrix (CM). An accompanying database resource called ChemGRID was developed to enable analysis, visualisation and downloads of all data. The CGM and CM datasets will facilitate the benchmarking of computational approaches for synergy prediction, as well as chemical structure-activity relationship models for anti-fungal drug discovery.

## Background & Summary

The network-based organization of biological systems suggests that combinations of small molecules will be needed to achieve therapeutic efficacy and selectivity for infectious disease, cancer and many other disorders^1,2^. Cellular networks are strongly buffered against loss of gene function, manifest as synthetic lethal genetic interactions^3^. A genetic interaction occurs when a phenotype caused by a mutation in one gene is exacerbated (or suppressed) by a mutation in another gene^4^. Genome-wide screens in the budding yeast *S. cerevisiae* have uncovered over 200,000 genetic interactions, in contrast to the ~1,000 essential genes^3,5^. Analogous to genetic interactions, combinations of chemicals that individually cause minimal phenotypes may exhibit greater than additive effects, termed synergism^2,6–9^. Notably, compounds that phenocopy the effect of mutations in non-essential genes may have no discernable effect on wild-type cells, but might inhibit growth in a given genetic context^2^. By definition, the biological activity of such compounds would not be detected in many high-throughput screens used in modern drug discovery. Compounds with such latent activities have been termed cryptagens or dark chemical matter^10,11^.

We recently generated a systematic chemical-genetic dataset in *S. cerevisiae* to allow the discovery and prediction of synergistic interactions between cryptagens that do not have obvious effects on cell proliferation on their own^11^. Various algorithmic approaches have been developed to predict synergistic compound combinations^1,12,13^. However, in most cases such predictions have been made on focused datasets and/or known chemical activities, which inherently constrains the development of general methods^14^. The dearth of fully factorial drug combination data matrices has hampered the systematic testing and comparisons of different predictive approaches^1^. To address this shortfall, we generated two large-scale data sets: a chemical-genetic matrix (CGM) of 356,500 pairwise chemical-gene interaction tests and a derived cryptagen matrix (CM) of 8,128 chemical-chemical interaction tests^11^. Based on this data, we developed a machine learning approach that integrates structural features of compounds with chemical-genetic interactions to predict compound synergism^11,15^. This systematic approach identified many novel synergistic anti-fungal combinations, many of which also exhibited species-selective effects against clinical isolates of pathogenic fungi^11^. The CM represents a benchmark dataset for the development and refinement of synergy prediction algorithms.

**Figure 1:**
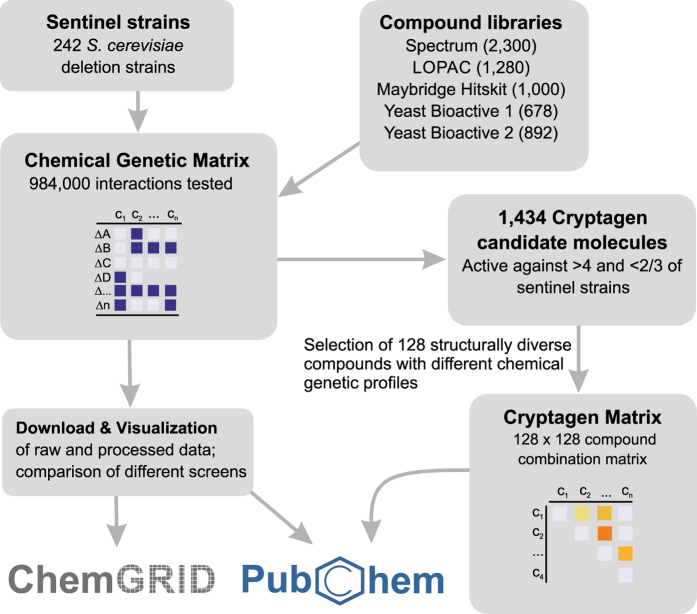
**Schematic overview of experimental workflow for CGM and CM dataset generation and data deposition.**

**Figure 2:**
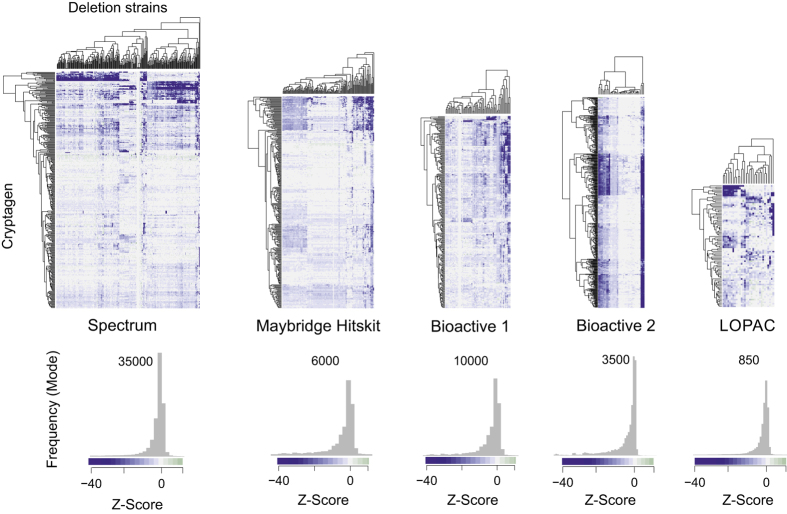
CGM heatmaps for activity (Z-score) of cryptagens contained in each of the five different compound libraries screened against the sentinel deletion strains. Corresponding histograms with Z-score distributions are shown below.

**Figure 3:**
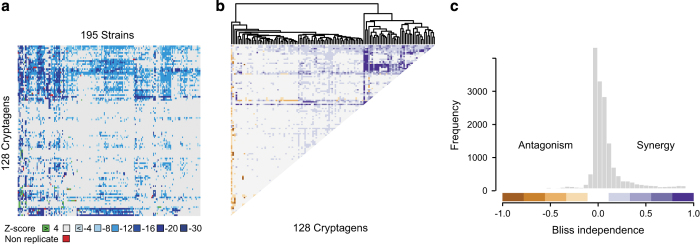
Representations of CM dataset. (**a**) Heatmap of growth inhibition for the 128 cryptagen compounds from the Microsource Spectrum library against 195 sentinel strains. (**b**) Heatmap of Bliss scores for all pairwise combinations of 128 cryptagen compounds. (**c**) Histogram of the Bliss score distribution.

**Figure 4:**
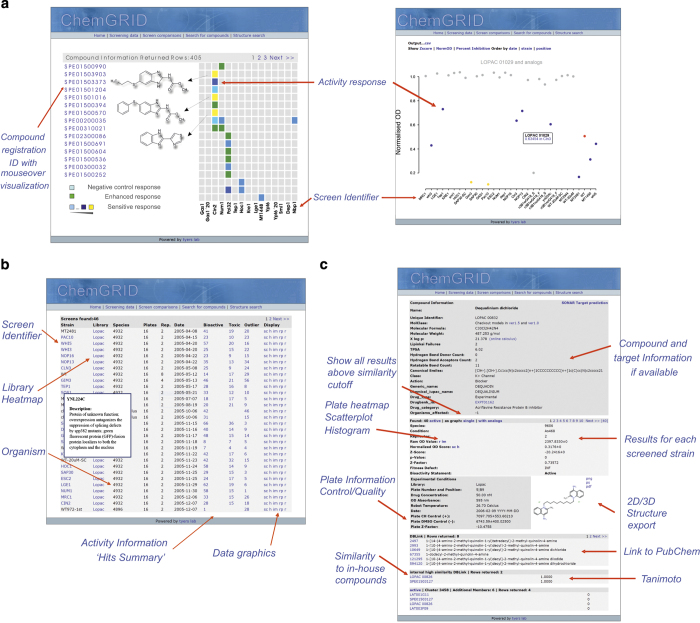
Screenshots of ChemGRID web portal functions. (**a**) Heatmap view of representative screens in ChemGRID (left) and accompanying data plot for a single screen (right). Mouseover functions allow interactive exploration of small molecule activities across different screens. (**b**) Overview page for the LOPAC screen component of the CGM. **(c)** The compound view page summarizes chemical properties, structure, and activity across all screens in the CGM, with automated internal links to similar compounds.

Here, we describe the CGM and CM datasets in detail to facilitate use of this data for synergy prediction by computational approaches. The original CGM was generated by screening 4,915 compounds drawn from four different chemical libraries (LOPAC, Maybridge Hitskit 1000, Spectrum Collection and an in-house collection called Bioactive 1). These libraries were screened against 195 diverse *S. cerevisiae* deletion strains, which we termed sentinel strains for their ability to detect otherwise hidden chemical activities^11^. The updated CGM described here is an extended version of the dataset reported previously: the number of sentinels has been increased from 195 to 242 yeast deletion strains and the cohort of chemical libraries has been expanded to include a second in-house collection of 892 compounds with bioactivity in yeast, termed Bioactive 2. This extended CGM dataset contains data for 5,518 unique compounds, 242 sentinel strains and duplicate measurements for 492,126 pairwise chemical-gene interaction tests, which represent an additional 135,626 duplicate interaction tests compared to the original CGM dataset (Figs 1,2; Table 1 (available online only)). As previously, we defined cryptagens as compounds that were active against more than 4 and less than 2/3 of tested sentinel strains. Out of the 5,518 compounds in the expanded CGM, 1,434 compounds were categorized as cryptagens (Table 2). From the original CGM dataset^11^, we selected a subset of 128 cryptagens that were used to generate a complete single concentration combination matrix, termed the cryptagen matrix (CM) (Fig. 3a). All 8,128 possible combinations between the 128 cryptagens were tested for synergy at 10 μM concentration for each compound in a drug pump-deficient *S. cerevisiae* strain (Fig. 3b). Bliss independence values were calculated for each compound pair in the CM dataset (see Methods for details). Independent dose-response surface (checkerboard) assays demonstrated a 65% confirmation rate of synergistic compound interactions from the CM dataset. The full CGM and CM datasets can be accessed at ChemGRID, a web portal that also houses a suite of tools that enable the interrogation and visualization of the chemical interaction datasets (Fig. 4). The CGM dataset and a detailed accompanying description of the yeast cell growth assay have been deposited at NCBI PubChem BioAssay (Data citations 1,2).

## Methods

The methods detailed below are expanded versions of descriptions in our related work^11^.

### Compound libraries used to generate the CGM

Compound libraries used were the LOPAC (Sigma), Maybridge Hitskit 1000 (Ryan Scientific) and the Spectrum Collection (MicroSource Discovery Systems Inc). We also screened two custom Yeast Bioactive Collections, termed Bioactive 1 and Bioactive 2, both of which were derived from screens of a 53,000 compound synthetic library (Ryan Scientific) in an *S. cerevisiae* cell proliferation assay at 10 μM (refs 16,17). The Bioactive 1 library contained 678 compounds that inhibited growth of a *pdr1*Δ̣*pdr3*Δ strain between 20% and 80%. Bioactive 2 contained 892 compounds that inhibited growth of a *pdr1*Δ̣*pdr3*Δ strain by at least 80%. Both of the Bioactive 1 and Bioactive 2 collections were selected to maximize chemical structural diversity compared to approved drugs listed by the World Health Organization (WHO). The approved drug list contains about 1,500 compounds that represent 50 Bemis-Murcko Fragments^18^. In contrast, the Bioactive 1 and Bioactive 2 collections contain 78 novel Bemis-Murcko scaffolds with uncharacterized modes of action. For all screens in the CGM, compound library stocks of 10 mM were diluted to 1 mM working stocks in DMSO in 96 well plates. Over the course of the study, the Spectrum Collection was re-purchased twice: the original library was called Spectrum03, and the two repurchased versions were called Spectrum05 and Spectrum08. As the composition of each release of this library differed somewhat, this non-redundancy resulted in 2,300 unique compounds in the combined Spectrum Collection used in this study. Library compositions and chemical structures are available at www.chemgrid.org.

### Yeast strains used to generate the CGM and the CM

The 242 different *S. cerevisiae* deletion strains used as sentinels to generate the CGM (Table 1 (available online only)) were obtained from the Euroscarf deletion collection and are isogenic to BY4741, which has the genotype *MAT*a *his3*Δ1 *leu2*Δ0 *met15*Δ0 *ura3*Δ0. For the CM, an isogenic *pdr1*Δ̣*pdr3*Δ; strain (MT2481) was generated from BY4741 using *PDR1*::*nat* and *PDR3*::*URA3* deletion cassettes.

### Generation of the chemical genetic matrix (CGM)

All screens were conducted in synthetic complete (SC) medium with 2% glucose. Yeast deletion strains (Table 1 (available online only)) were seeded at 50,000 cells per well from fresh overnight cultures in a screening volume of 100 μl in 96 well plates. 2 μl of 1 mM compound stock was added to each well for a final compound concentration of 20 μM. Screens were conducted in technical duplicates using a Biomek FX liquid handling workstation with an integrated stacker carousel. 10 μM cycloheximide positive controls and DMSO solvent-only controls were set up in columns 1 and 12 of each 96 well plate. All plates were incubated at 30 °C without shaking for approximately 18 h or until solvent-treated control cultures were saturated. Cultures were then resuspended by shaking on the robotic platform before reading OD_600_ values on either Tecan Sunrise or Tecan M1000 plate readers^19^. Biological repeats were generated every 8 months over the course of the study for both the *pdr1*Δ*pdr3*Δ strain and a wild-type *S. cerevisiae* strain (BY4741) to ensure consistency of compound activity and data reproducibility.

### Statistical analysis of the CGM data

All screening data was subjected to the following analysis workflow:

1. LOWESS regression was used to correct spatial effects on growth across all plates for all screens performed with the LOPAC, Maybridge Hitskit 1000 and Spectrum Collection libraries, and for all but seven screens with the Bioactive 1 library. An empirically estimated sliding window of 1/3 was used and data normalization was based on the plate median. The LOWESS normalization method effectively removed variable plate edge effects within the non-active fraction of compounds, which sometimes occurred when plates were read at late time points.
2. For the seven Bioactive 1 screens with higher hit rates, and for all Bioactive 2 screens, the data was not LOWESS corrected but was instead normalized to DMSO controls. The Bioactive 1 library was selected for enrichment of compounds with moderate bioactivity against yeast, whereas the Bioactive 2 library was selected for compounds with strong bioactivity.
3. Median-normalization was applied to all plates and experiments.
4. Z-scores for growth inhibition were calculated based on the median and the interquartile range (IQR) by fitting a normal distribution with N(1,IQR) to the experimental data. We note that the IQR was intentionally chosen as a conservative estimate of variance to reduce the risk of false positives among the weakly active compounds in the screen. This approach slightly underestimates the significance of the Z-scores. In addition, Z-scores, per cent inhibition and normalized OD values were calculated for manual data validation.
5. Data points with high variation between replicates with growth inhibition up to 30% (>3 MAD) were flagged as inconsistent outliers and removed from further analysis.
6. Classification of compounds into ‘active’ and ‘enhanced growth’ was based on Z-score cut-offs. Compounds with Z-scores<−4 were classified as ‘active’, i.e., with reduced OD values compared to the negative solvent-only control. Positive Z-scores >4 were classified as ‘enhanced growth’, i.e., with OD values greater than the negative control.

All raw and processed CGM data are available online at http://chemgrid.org/cgm and from NCBI PubChem BioAssay (Data citation 1).

### Selection of cryptagen compounds for the cryptagen matrix (CM)

From the CGM dataset, we identified 1,434 cryptagen compounds. These compounds inhibited growth of at least four and less than two-thirds of the yeast deletion strains. Each of the four chemical libraries yielded cryptagen compounds: the LOPAC at a hit rate of 5%, the Maybridge Hitskit at 27%, the Spectrum Collection at 18%, Bioactive 1 at 10% and Bioactive 2 at 23%. For the generation of the CM, cryptagens were selected from the Microsource Spectrum Collection based on activities against 58 different sentinel strains (Table 1 (available online only)). All cryptagens from the Spectrum Collection were clustered (average linkage hierarchical clustering) based on chemical structure and a structurally diverse set of 128 compounds with diverse chemical-genetic profiles was selected for the generation of the CM.

### Generation of the CM

The 128 Spectrum compounds used for the CM were resupplied from MicroSource Discovery Systems Inc. (Groton, CT). Compounds were diluted to a stock concentration of 0.5 mM and arrayed in two 96 well plates. The 128×128 matrix was generated at 10 μM per compound in duplicate experiments (i.e, biological replicates) using a *pdr1*Δ̣*pdr3*Δ *S. cerevisiae* strain in 96 well plates. Yeast cultures were seeded at 50,000 cells per well in synthetic complete (SC) medium with 2% glucose at a screening volume of 100 μl. For the combination screens, 2 μl of 0.5 mM compound stock was added for both compounds for a final concentration of 10 μM per compound. The 128 compounds were also screened in combination with 2 μl DMSO to obtain growth inhibition data for each compound alone for compound interaction calculations. DMSO solvent-only controls were set up in columns 1 and 12 as well as four wells with a 10 μM cycloheximide positive control for complete growth inhibition in column 12. Plates were incubated for approximately 18 h or until solvent control cultures were saturated at 30 °C without shaking. Cultures were then resuspended by shaking on the robotic platform before reading OD_600_ values on either Tecan Sunrise or Tecan M1000 plate readers^19^.

### Analysis of CM data

OD_600_ measurements for the CM were normalized to DMSO controls and data was averaged between the biological replicates. Bliss independence^20^ for compounds X and Y was calculated using the equation *I*_*xy*_=*I*_*x*_+*I*_*y*_−(*I*_*x*_×*I*_*y*_) where *I*_*x*_ and *I*_*y*_ correspond to growth inhibition in the presence of 10 μM compound X and Y, respectively. The expected growth inhibition for combination treatment with X and Y was compared to the actual growth inhibition observed in the CM to obtain the Bliss independence values. Bliss independence values within 90% density kernel fit represented additive effects. Based on the density kernel density estimation, Bliss independence values larger than 0.25 represented synergism and values below −0.18, antagonism. All raw and processed CM data are available online at http://chemgrid.org/cgm and from NCBI PubChem BioAssay (Data citation 2).

### Note about the Bliss independence model

The Bliss independence model is based on testing two compounds and the pairwise combination at single concentrations^20^. The Bliss model does not account for possible non-linear concentration effects of either drug, which would instead require assessment over a two dimensional dose-response surface to determine Loewe additivity^21^, also sometimes calculated as the fractional inhibitory concentration index or FICI^6^. The Loewe additivity model requires extensive single and combination drug inhibition measurements that are not practical for large-scale surveys of drug combinations. Therefore, our initial estimates for synergism in the CM relied on the Bliss independence model, as calculated from single concentrations for each drug and the pairwise combination, using the equation *I*_*xy*_=*I*_*x*_+*I*_*y*_−(*I*_*x*_ *I*_*y*_)^6^. The Bliss independence model for growth inhibition is equivalent to the multiplicative fitness model used to quantify genetic interactions. If two genes A and B do not interact, the expected fitness *F* of the double deletion strain is *F*_*ΔAΔB*_ *=F*_*ΔA*_×*F*_*ΔB*_ where *F*_*ΔA*_ and *F*_*ΔB*_ represent the fitness defects of the two single deletion strains. Replacing fitness *F* with growth inhibition (*F=*1−*I*) in the genetic model yields: *1*−*I*_*ΔAΔB*_ *=(*1−*I*_*ΔA*_ *)×(*1−*I*_*ΔB*_) which can be simplified to 1−*I*_*ΔAΔB*_ *=*1−*I*_*ΔB*_−*I*_*ΔA*_+*I*_*ΔA*_×*I*_*ΔB*_. Subtracting ‘1’ and multiplication with (*–*1) yields the Bliss independence formula for growth inhibition: *I*_*ΔAΔB*_ *=I*_*ΔA*_+*I*_*ΔB*_−*I*_*ΔA*_×*I*_*ΔB*_. For example, if two single deletion strains, *ΔA* and *ΔB*, have fitness values of 0.6 and 0.8, compared to a wild type fitness of 1.0, the expected fitness of the double deletion strain *ΔAΔB* is F_ΔAΔB_=0.6 * 0.8=0.48, corresponding to a growth inhibition of 52%. Similarly, if two small molecules A and B inhibit growth by 40 and 20%, their expected combined growth inhibition based on the Bliss independence model is I_AB_=0.4+0.2−(0.5×0.2)=0.6–0.08=0.52. Thus, if compounds A and B do not interact (i.e., have an additive effect), we expect a growth inhibition of 52% for this compound combination. Two genes are said to display a negative genetic interaction when the observed fitness of the double deletion strain is lower than expected. Similarly, synergy between two small molecules is observed when growth inhibition in response to the combination treatment is higher than expected.

### Code availability

The data analysis procedures for the CGM were implemented in R using the additional packages RMySQL, outliers, matlab, amap, RSvgDevice and RSvgTipsDevice. We developed ChemGRID (http://chemgrid.org/cgm) as a webportal for the upload, processing and visualisation of chemical-genetic screen data (Fig. 4a,b) using PHP, PEAR, Perl and MySQL. The cheminformatics functionalities to register structural information were implemented with Python and FROWNS, PerlMol and MolDB4 (ref. 22). All code for data analysis is available at https://github.com/jwildenhain/chemical-genetic-matrix.

## Data Records

### Data record 1—chemical genetic matrix (CGM)

The chemical genetic data set of five different compound libraries screened against different subsets of 242 different *S. cerevisiae* deletion strains. In total, 492,126 chemical-genetic interaction tests (i.e., 984,252 independent measurements since each test was performed in duplicate) between 5,518 compounds and 242 *S. cerevisiae* deletion strains are represented in the CGM dataset. All raw and processed CGM data can be downloaded and visualized using ChemGRID (http://chemgrid.org/cgm) and are available from NCBI PubChem BioAssay (Data citation 1).

### Data record 2—cryptagen matrix (CM)

The raw data as well as the Bliss independence values for all 8,128 pairwise combinations of 128 cryptagen compounds are available from NCBI PubChem BioAssay (Data citation 2). Bliss independence value calculations are described in detail in the Method section above.

## Technical Validation

### In-plate controls

For the CGM and the CM, each screening plate contained DMSO solvent-only controls as well as cycloheximide positive controls to ensure that inoculum preparation, compound pipetting and screening conditions were not compromised. DMSO solvent-only controls were used for data normalization to account for plate-to-plate variation within each screen and for variation between different screens.

### Technical and biological replicates

**Figure 5:**
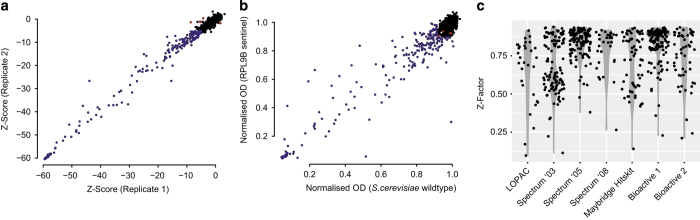
Technical validation of CGM data. (**a**) Technical replicates of *S. cerevisiae* wild type screen data. (**b**) Scatter plot of *S. cerevisiae* wild type versus *rbl9Δ* screen data. (**c**) Z-factor distributions for each individual compound library screened in the CGM.

During generation of the CGM, each *S. cerevisiae* deletion strain was screened in a technical or biological replicate. In addition, we repeatedly screened the compound collections against the *pdr1*Δ*pdr3*Δ strain and a wild-type *S. cerevisiae* strain (BY4741) to ensure data reproducibility (Fig. 5a,b).

### Z-factor

For each library screen, Z-factors were calculated to ensure quality of the screening data (Fig. 5c).

### Quality filter

Small molecules that did not reproduce between replicate screens were filtered out and not used for further analysis of the CGM data.

### Biological replicates

Every compound combination was tested in biological replicate on different days.

### Surface dose-response (checkerboard) assays

A set of 75 combinations from the CM was tested in 4×4 checkerboard assays. 40 of these combinations initially showed synergistic interactions and 35 did not. This checkerboard analysis confirmed 23 interactions as true positives and 26 as true negatives, with 9 false negatives and 17 false positives. Overall, these confirmatory assays resulted in a 65% confirmation rate of synergistic compound interactions from the CM dataset.

## Usage Notes

### Dataset applications

The linked CGM chemical-genetic interaction and CM chemical-chemical interaction datasets were designed to serve as resources for computational predictions of chemical synergism, and were used in our original study to predict synergistic antifungal combinations^11^. To our knowledge, the CM is the first unbiased large-scale benchmark dataset for chemical-chemical interactions. The CGM and CM datasets can also be used to derive chemical structure-activity relationships for anti-fungal drug discovery. Furthermore, the CGM data can be used to infer compound mode of action^11,13,23,24^.

### ChemGRID functionality

The ChemGRID webportal is designed to allow the upload, processing and visualisation of chemical-genetic screen data (Fig. 4a,b). All of the raw and processed CGM data is available on ChemGRID and the dataset for each individual chemical library can be browsed, visualized and downloaded. Screens against different *S. cerevisiae* deletion strains with the same compound collection can be compared and a mouse-over functionality allows for interactive data exploration, including chemical structures and properties. Scatterplots can be generated to compare data from different screens, for example between a wild type and a specific deletion strain. Compounds can also be searched by name and chemical structure. Data for single compounds across different screens can be retrieved and viewed on a summary page together with chemical properties, structures, and links to similar compounds (Fig. 4c). ChemGRID also provides linkouts to other webportals for drug target and compound activity information, including Drugbank^25^, PubChem^26^, MolClass^15^ and BioGRID^5^.

## Additional Information

**How to cite this article**: Wildenhain, J. *et al.* Systematic chemical-genetic and chemical-chemical interaction datasets for prediction of compound synergism. *Sci. Data* 3:160095 doi: 10.1038/sdata.2016.95 (2016).

**Publisher’s note**: Springer Nature remains neutral with regard to jurisdictional claims in published maps and institutional affiliations.

## Supplementary Material



## Figures and Tables

**Table 1:** Strains screened against each library in the CGM

**ORF**	**Gene Name**	**LOPAC**	**Maybridge HitsKit 1000**	**Spectrum**	**Bioactive 1**	**Bioactive 2**	**Use in CM**	**Synonym**	**Description** *	**Phenotype information** *	**SGD ID** *
YLR027C	AAT2			**+**				ASP5	Cytosolic aspartate aminotransferase, involved in nitrogen metabolism; localizes to peroxisomes in oleate-grown cells	Null mutant is viable in S288C, inviable in Sigma1278b	S000004017
YCR088W	ABP1			**+**					Actin-binding protein of the cortical actin cytoskeleton, important for activation of the Arp2/3 complex that plays a key role actin in cytoskeleton organization	Null mutant is viable, has abnormalities in actin cytoskeleton morphology	S000000684
YDR226W	ADK1†				**+**			AKY1|AKY2	Adenylate kinase, required for purine metabolism; localized to the cytoplasm and the mitochondria; lacks cleavable signal sequence	Null mutant is viable. displays slow growth and inability to utilize non-fermentable carbon sources	S000002634
YNR030W	ALG12		**+**					ECM39	Alpha-1,6-mannosyltransferase localized to the ER; responsible for the addition of the alpha-1,6 mannose to dolichol-linked Man7GlcNAc2, acts in the dolichol pathway for N-glycosylation	Null mutant is viable, confers increased resistance to oxidative stress	S000005313
YLR102C	APC9			**+**					Subunit of the Anaphase-Promoting Complex/Cyclosome (APC/C), which is a ubiquitin-protein ligase required for degradation of anaphase inhibitors, including mitotic cyclins, during the metaphase/anaphase transition	Null mutant is viable, shows delay in entry into anaphase at 37 C	S000004092
YLR370C	ARC18			**+**					Subunit of the ARP2/3 complex, which is required for the motility and integrity of cortical actin patches	Null mutant is viable, fails to localize actin patches, accumulates high amounts of chitin in cell wall, and shows defects in mitochondrial movement	S000004362
YHR013C	ARD1			**+**					Subunit of the N-terminal acetyltransferase NatA (Nat1p, Ard1p, Nat5p); N-terminally acetylates many proteins, which influences multiple processes such as the cell cycle, heat-shock resistance, mating, sporulation, and telomeric silencing	Null mutant is viable, displays decreased pheromone sensitivity, reduced mating efficiency and sporulation	S000001055
YDL192W	ARF1			**+**					ADP-ribosylation factor, GTPase of the Ras superfamily involved in regulation of coated formation vesicles in intracellular trafficking within the Golgi; functionally interchangeable with Arf2p	Null mutant is viable, shows slow growth, cold sensitivity and sensitivity to normally sublethal concentrations of fluoride ion in the medium.	S000002351
YNL020C	ARK1				**+**		**+**		Serine/threonine protein kinase involved in regulation of the cortical actin cytoskeleton; involved in control of endocytosis	Null mutant is viable, shows slight delocalisation of actin cytoskeleton	S000004965
YPL051W	ARL3			**+**					GTPase of the Ras superfamily required to recruit Arl1p to the Golgi; similar to ADP-ribosylation factor	Null mutant is viable, displays cold-sensitive growth	S000005972
YHR129C	ARP1			**+**			**+**	ACT5	Actin-related protein of the dynactin complex; required for spindle orientation and nuclear migration; putative ortholog of mammalian centractin	Null mutant is viable, both null mutations and overexpression lead to defects in spindle orientation and nuclear migration	S000001171
YLR085C	ARP6		**+**				**+**		Nuclear actin-related protein involved in chromatin remodeling, component of chromatin-remodeling enzyme complexes	Null mutant is viable, disorganized actin filaments and abnormal vacuolar morphology	S000004075
YDR101C	ARX1			**+**					Protein associated with the ribosomal export complex	Null mutant is viable, modest nuclear retention of 60S ribosomal subunits	S000002508
YJL115W	ASF1			**+**				CIA1	Nucleosome assembly factor, involved in chromatin assembly after DNA replication, anti-silencing protein that causes derepression of silent loci when overexpressed	Null mutant is viable, delayed cell cycle progression through metaphase, defect in telomere localization, decreased silencing, shortened lifespan	S000003651
YPL078C	ATP4			**+**				LPF7	Subunit b of the stator stalk of mitochondrial F1F0 ATP synthase, which is a large, evolutionarily conserved enzyme complex required for ATP synthesis	Null mutant is viable, oxidative phosphorylation deficient, unable to grow on glycerol	S000005999
YOL078W	AVO1†				**+**				Component of a membrane-bound complex containing the Tor2p kinase and other proteins, which may have a role in regulation of cell growth	Null mutant is inviable, depolarized actin cytoskeleton	S000005438
YJL095W	BCK1			**+++**	**+**	**+**	**+**	LAS3|SAP3|SLK1|SSP31	Mitogen-activated protein (MAP) kinase kinase kinase acting in the protein kinase C signaling pathway, which controls cell integrity; upon activation by Pkc1p phosphorylates downstream kinases Mkk1p and Mkk2p	Null mutant is viable, temperature-sensitive cell wall defect	S000003631
YER167W	BCK2			**+**			**+**	CTR7	Protein rich in serine and threonine residues, overproduction suppresses pkc1 mutation, activates G1/S transcription	Null mutant is viable, increased cell size and pheromone sensitivity	S000000969
YER155C	BEM2			**++**			**+**	IPL2|SUP9|TSL1	Rho GTPase activating protein (RhoGAP) involved in the control of cytoskeleton organization and cellular morphogenesis; required for bud emergence	Null mutant is viable, randomized bud-site selection and defective bud emergence	S000000957
YCL029C	BIK1			**+**				ARM5|PAC14	Microtubule-associated protein, component of the interface between microtubules and kinetochore, involved in sister chromatid separation; essential in polyploid cells but not in haploid or diploid cells; ortholog of mammalian CLIP-170	Null mutant is viable, bilateral defects in karyogamy	S000000534
YER016W	BIM1			**+**				EB1|YEB1	Microtubule-binding protein that together with Kar9p makes up the cortical microtubule capture site and delays the exit from mitosis when the spindle is oriented abnormally	Null mutant is viable, cold sensitivie, benomyl supersensitive, aberrant microtubule morphology	S000000818
YNL271C	BNI1			**+**				PPF3|SHE5	Formin, nucleates the formation of linear actin filaments, involved in cell processes such as budding and mitotic spindle orientation which require the formation of polarized actin cables, functionally redundant with BNR1	Null mutant is viable, bni1 bnr1 double deletion mutant is temperature sensitive, deficient in bud emergence, random distribution of cortical actin patches	S000005215
YNL233W	BNI4			**+**					Targeting subunit for Glc7p protein phosphatase, localized to the bud neck, required for localization of chitin synthase III to the bud neck via interaction with the chitin synthase III regulatory subunit Skt5p	Null mutant is viable, shows delocalized chitin, elongated buds, enlarged bud necks	S000005177
YDL074C	BRE1			**+**			**+**		E3 ubiquitin ligase for Rad6p, required for the ubiquitination of histone H2B, recruitment of Rad6p to promoter chromatin and subsequent methylation of histone H3 (on L4 and L79), contains RING finger domain	Null mutant is viable, sensitive to brefeldin A	S000002232
YFL029C	CAK1†				**+**		**+**	CIV1	Cyclin-dependent kinase-activating kinase required for passage through the cell cycle, phosphorylates and activates Cdc28p; nucleotide-binding pocket differs significantly from those of most other protein kinases	Null mutant is inviable, temperature-sensitive mutant confers a G2 delay	S000001865
YNL161W	CBK1†				**+**				Serine/threonine protein kinase that regulates cell morphogenesis pathways; involved in cell wall biosynthesis, apical growth, proper mating projection morphology, bipolar bud site selection in diploid cells, and cell separation	Null mutation is inviable, defect in morphogenesis and cell separation	S000005105
YPR025C	CCL1†				**+**				Cyclin associated with protein kinase Kin28p, which is the TFIIH-associated carboxy-terminal domain (CTD) kinase involved in transcription initiation at RNA polymerase II promoters	Null mutant is inviable, reduced function mutants show increased competitive fitness	S000006229
YAL021C	CCR4			**+**				FUN27|NUT21	Component of the CCR4-NOT transcriptional complex, which is involved in regulation of gene expression; component of the major cytoplasmic deadenylase, which is involved in mRNA poly(A) tail shortening	Null mutant is viable, temperature sensitive growth on nonfermentative medium, sensitive to many drugs	S000000019
YFR028C	CDC14†				**+**			OAF3	Protein phosphatase required for mitotic exit; located in the nucleolus until liberated by the FEAR and Mitotic Exit Network in anaphase, enabling it to act on key substrates to effect a decrease in CDK/B-cyclin activity and mitotic exit	Null mutant is inviable; temperature sensitive mutant arrests at late anaphase	S000001924
YAR019C	CDC15†				**+**			LYT1	Protein kinase of the Mitotic Exit Network that is localized to the spindle pole bodies at late anaphase; promotes mitotic exit by directly switching on the kinase activity of Dbf2p	Null mutant is inviable, temperature sensitive mutant arrests at late anaphase	S000000072
YBR160W	CDC28†				**+**			CDK1|HSL5|SRM5	Catalytic subunit of the main cell cycle cyclin-dependent kinase (CDK); alternately associates with G1 cyclins (CLNs) and G2/M cyclins (CLBs) which direct the CDK to specific substrates	Null mutant is inviable, temperature sensitive mutants arrest at G1/S and G2/M	S000000364
YDR054C	CDC34†				**+**			DNA6|UBC3	Ubiquitin-conjugating enzyme or E2; together with Skp1p, Rbx1p, Cdc53p, and an F-box protein, forms a ubiquitin-protein ligase called the SCF complex which regulates cell cycle progression by targeting key substrates for degradation	Null mutant is inviable, temperature sensitive mutant arrests at G1/S with multiple buds	S000002461
YFL009W	CDC4†				**+**				F-box protein required for G1/S and G2/M transition, associates with Skp1p and Cdc53p to form a complex, SCFCdc4, which acts as ubiquitin-protein ligase directing ubiquitination of the phosphorylated CDK inhibitor Sic1p	Null mutant is inviable, temperature sensitive mutant arrests at G1/S with multiple buds	S000001885
YMR001C	CDC5†				**+**			MSD2|PKX2	Polo-like kinase with similarity to Xenopus Plx1 and S. pombe Plo1p; found at bud neck, nucleus and SPBs; has multiple functions in mitosis and cytokinesis through phosphorylation of substrates; may be a Cdc28p substrate	Null mutant is inviable, temperature sensitive mutant arrests in late anaphase	S000004603
YDL132W	CDC53†				**+**				Cullin, structural protein of SCF complexes (which also contain Skp1p, Cdc34p, and an F-box protein) involved in ubiquitination; SCF promotes the G1-S transition by targeting G1 cyclins and the Cln-CDK inhibitor Sic1p for degradation	Null mutant is inviable, temperature sensitive mutant arrests at G1/S with multiple buds	S000002290
YDL017W	CDC7†				**+**			LSD6|SAS1	DDK (Dbf4-dependent kinase) catalytic subunit required for firing origins and replication fork progression in mitosis through phosphorylation of Mcm2-7p complexes and Cdc45p; kinase activity correlates with cyclical DBF4 expression	Null mutant is inviable, temperature sensitive mutant arrests at G1/S	S000002175
YLR418C	CDC73			**+**					Constituent of Paf1 complex with RNA polymerase II, Paf1p, Hpr1p, Ctr9, Leo1, Rtf1 and Ccr4p, distinct from Srb-containing Pol II complexes; required for expression of certain genes, modification of some histones, and telomere maintenance	Null mutant is viable, temperature sensitive and cold sensitive	S000004410
YBR023C	CHS3			**+**				CAL1|CSD2|DIT101|KTI2	Chitin synthase III, catalyzes the transfer of N-acetylglucosamine (GlcNAc) to chitin; required for synthesis of the majority of cell wall chitin, the chitin ring during bud emergence, and spore wall chitosan	Null mutant is viable, exhibits cell wall defects	S000000227
YLR330W	CHS5			**+**			**+**	CAL3	Protein of unknown function, involved in chitin biosynthesis by regulating Chs3p localization, also involved in cell fusion during mating	Null mutant is viable, exhibits cell wall defects	S000004322
YHR142W	CHS7			**+**					Protein of unknown function, involved in chitin biosynthesis by regulating Chs3p export from the ER	Null mutant is viable, exhibits cell wall defects	S000001184
YPL241C	CIN2	**+**	**+**	**+**			**+**		Tubulin folding factor C (putative) involved in beta-tubulin (Tub2p) folding; isolated as mutant with increased chromosome loss and sensitivity to benomyl	Null mutant is viable, sensitive to benomyl, cold sensitive, increased rate of chromosome loss	S000006162
YEL061C	CIN8		**+**	**++**			**+**	KSL2|SDS15	Kinesin motor protein involved in mitotic spindle assembly and chromosome segregation	Null mutant is viable, sensitive to benomyl, increased rate of chromosome loss	S000000787
YOR061W	CKA2			**+**	**+**		**+**	YOR29-12	Alpha- catalytic subunit of casein kinase 2, a Ser/Thr protein kinase with roles in cell growth and proliferation; the holoenzyme also contains CKA1, CKB1 and CKB2, the many substrates include transcription factors and all RNA polymerases	Null mutant is viable, resistant to many drugs, increased lifespan	S000005587
YLR133W	CKI1				**+**				Choline kinase, catalyzes the first step in the CDP-choline pathway phosphatidylcholine synthesis (Kennedy pathway); mRNA expression is regulated by inositol and choline, enzyme activity is stimulated by phosphorylation by protein kinase	Null mutant is viable, resistant to many drugs, increased lifespan	S000004123
YBR135W	CKS1†				**+**				Subunit of the Cdc28 protein kinase, required for mitotic proteolysis, may also be involved in the proteolysis of the G1 cyclins	Null mutant is inviable, temperature sensitive mutant arrests at G1/S	S000000339
YNL298W	CLA4		**+**	**+**				ERC10	Cdc42p activated signal transducing kinase of the PAK (p21-activated kinase) family, involved in septin ring assembly and cytokinesis; directly phosphorylates septins Cdc3p and Cdc10p; other yeast PAK family members are Ste20p and Skm1p	Null mutant is viable, cytokinesis defect	S000005242
YPR119W	CLB2			**+**					B-type cyclin involved in cell cycle progression; activates Cdc28p to promote the transition from G2 to M phase; accumulates during G2 and M, then targeted via a destruction box motif for ubiquitin-mediated degradation by the proteasome	Null mutant is viable, delayed G2/M progression, increased cell size	S000006323
YPL256C	CLN2			**++**	**+**	**+**			G1 cyclin involved in regulation of the cell cycle; activates Cdc28p kinase to promote the G1 to S phase transition; late G1 specific expression depends on transcription factor complexes, MBF (Swi6p-Mbp1p) and SBF (Swi6p-Swi4p)	Null mutant is viable, delayed G1/S progression, increased cell size	S000006177
YAL040C	CLN3	**+**					**+**	DAF1|FUN10|WHI1	G1 cyclin involved in cell cycle progression; activates Cdc28p kinase to promote the G1 to S phase transition; plays a role in regulating transcription of the other G1 cyclins, CLN1 and CLN2; regulated by phosphorylation and proteolysis	Null mutant is viable, delayed G1/S transition, increased cell size	S000000038
YKL190W	CNB1		**+**	**+**			**+**	CRV1|YCN2	Calcineurin B; the regulatory subunit of calcineurin, a Ca++/calmodulin-regulated protein phosphatase which regulates Crz1p (a stress-response transcription factor), the other calcineurin subunit is encoded by CNA1 and/or CMP1	Null mutant is viable, Li+ and Na+ sensitive	S000001673
YOR316C	COT1			**+**					Vacuolar transporter that mediates zinc transport into the vacuole; overexpression confers resistance to cobalt and rhodium	Null mutant is viable, increased sensitivity to cobalt	S000005843
YMR078C	CTF18			**+**				CHL12	Subunit of a complex with Ctf8p that shares some subunits with Replication Factor C and is required for sister chromatid cohesion; may have overlapping functions with Rad24p in the DNA damage replication checkpoint	Null mutant is viable, increased mitotic recombination, slow growth, cold sensitivity	S000004683
YPR135W	CTF4		**+**					CHL15|POB1	Chromatin-associated protein, required for sister chromatid cohesion; interacts with DNA polymerase alpha (Pol1p) and may link DNA synthesis to sister chromatid cohesion	Null mutant is viable, increased chromosome loss rate, and mitotic recombination	S000006339
YML070W	DAK1				**+**				Dihydroxyacetone kinase, required for detoxification of dihydroxyacetone (DHA); involved in stress adaptation	Null mutant is viable, sensitive to dihydroxyacetone	S000004535
YGR092W	DBF2				**+**		**+**		Ser/Thr kinase involved in transcription and stress response; functions as part of a network of genes in exit from mitosis; localization is cell cycle regulated; activated by Cdc15p during the exit from mitosis	Null mutant is viable, dbf1 dbf20 double mutant arrests in late telophase	S000003324
YPR111W	DBF20				**+**				Ser/Thr kinase involved in late nuclear division, one of the mitotic exit network (MEN) proteins; necessary for the execution of cytokinesis	Null mutant is viable, dbf1 dbf20 double mutant arrests in late telophase	S000006315
YDR052C	DBF4†				**+**			DNA52|LSD7	Regulatory subunit of Cdc7p-Dbf4p kinase complex, required for Cdc7p kinase activity and initiation of DNA replication; phosphorylates the Mcm2-7 family of proteins; cell cycle regulated	Null mutant is inviable; temperature sensitive mutant arrests at G1/S	S000002459
YGL078C	DBP3			**+**					Putative ATP-dependent RNA helicase of the DEAD-box family involved in ribosomal biogenesis	Null mutant is viable, severe growth defect, thermotolerant	S000003046
YCL016C	DCC1			**+**					Subunit of a complex with Ctf8p and Ctf18p that shares some components with Replication Factor C, required for sister chromatid cohesion and telomere length maintenance	Null mutant is viable, benomyl sensitive, increased chromosome loss rate	S000000521
YIR030C	DCG1			**+**					Protein of unknown function, expression is sensitive to nitrogen catabolite repression and regulated by Dal80p; contains transmembrane domain	Null mutant is viable, abnormal vaculoar morphology	S000001469
YAL013W	DEP1	**+**	**+**	**+**			**+**	FUN54	Transcriptional modulator involved in regulation of structural phospholipid biosynthesis genes and metabolically unrelated genes, as well as maintenance of telomeres, mating efficiency, and sporulation	Null mutant is viable, increased silencing, increased cell size	S000000011
YOR080W	DIA2			**+**				YOR29-31	Origin-binding F-box protein that forms an SCF ubiquitin ligase complex with Skp1p and Cdc53p; plays a role in DNA replication, involved in invasive and pseudohyphal growth	Null mutant is viable, sensitive to DNA damage, increased chromsome loss rate, anaphase delay	S000005606
YLL001W	DNM1			**+**					Dynamin-related GTPase required for mitochondrial fission and the maintenance of mitochondrial morphology, assembles on the cytoplasmic face of mitochondrial tubules at sites at which division will occur; also participates in endocytosis	Null mutant is viable, reduced autophagy, increased lifespan; aberrant mitochondrial and peroxisomal morphology	S000003924
YDR069C	DOA4			**+**				DOS1|MUT4|NPI2|SSV7|UBP4	Ubiquitin hydrolase, required for recycling ubiquitin from proteasome-bound ubiquitinated intermediates, acts at the late endosome/prevacuolar compartment to recover ubiquitin from ubiquitinated membrane proteins en route to the vacuole	Null mutant is viable, sensitive to many drugs	S000002476
YGL240W	DOC1			**+**				APC10	Processivity factor required for the ubiquitination activity of the anaphase promoting complex (APC), mediates the activity of the APC by contributing to substrate recognition; involved in cyclin proteolysis	Null mutant is viable, delayed metaphase to anaphase transition, increased chromsome loss rate	S000003209
YAL026C	DRS2			**+**				FUN38|SWA3	Integral membrane Ca(2+)-ATPase involved in aminophospholipid translocation; required to form a specific class of secretory vesicles that accumulate upon actin cytoskeleton disruption; mutation affects maturation of the 18S rRNA	Null mutant is viable, sensitive to cell wall-damaging agents, defective protein transport through the Golgi	S000000024
YDL101C	DUN1		**+**	**+**	**+**		**+**		Cell-cycle checkpoint serine-threonine kinase required for DNA damage-induced transcription of certain target genes, phosphorylation of Rad55p and Sml1p, and transient G2/M arrest after DNA damage; also regulates postreplicative DNA repair	Null mutant is viable, defective in DNA damage response	S000002259
YKL160W	ELF1			**+**					Transcription elongation factor that contains a conserved zinc finger domain; implicated in the maintenance of proper chromatin structure in actively transcribed regions; deletion inhibits Brome mosaic virus (BMV) gene expression	Null mutant is viable, decreased stress resistance	S000001643
YOR144C	ELG1			**+**				RTT110	Protein required for S phase progression and telomere homeostasis, forms an alternative replication factor C complex important for DNA replication and genome integrity; mutants are sensitive to DNA damage	Null mutant is viable, increased chromosome loss rate, defective DNA damage response	S000005670
YKL048C	ELM1				**+**			LDB9	Serine/threonine protein kinase that regulates cellular morphogenesis, septin behavior, and cytokinesis; required for the regulation of other kinases; forms part of the bud neck ring	Null mutant is viable, abnormal morphology, delayed G2/M transition	S000001531
YMR219W	ESC1		**+**						Protein localized to the nuclear periphery, involved in telomeric silencing; interacts with PAD4-domain of Sir4p	Null mutant is viable, defective telomeric silencing	S000004832
YDR363W	ESC2	**+**					**+**		Protein involved in mating-type locus silencing, interacts with Sir2p; probably functions to recruit or stabilize Sir proteins	Null mutant is viable, defective telomeric silencing, increased chromosome loss rate	S000002771
YER015W	FAA2			**+**				FAM1	Long chain fatty acyl-CoA synthetase; accepts a wider range of acyl chain lengths than Faa1p, preferring C9:0-C13:0; involved in the activation of endogenous pools of fatty acids	Null mutant is viable	S000000817
YFR019W	FAB1				**+**			SVL7	1-phosphatidylinositol-3-phosphate 5-kinase; vacuolar membrane kinase that generates phosphatidylinositol (3,5)P2, which is involved in vacuolar sorting and homeostasis	Null mutant is viable, temperature sensitive, defective vacuolar transport and morphology	S000001915
YJL157C	FAR1				**+**				Cyclin-dependent kinase inhibitor that mediates cell cycle arrest in response to pheromone; also forms a complex with Cdc24p, Ste4p, and Ste18p that may specify the direction of polarized growth during mating; potential Cdc28p substrate	Null mutant is viable, fails to arrest in G1 upon pheromone exposure	S000003693
YMR277W	FCP1†				**+**				Carboxy-terminal domain (CTD) phosphatase, essential for dephosphorylation of the repeated C-terminal domain of the RNA polymerase II large subunit (Rpo21p)	Null mutant is inviable	S000004890
YMR058W	FET3			**+**					Ferro-O2-oxidoreductase required for high-affinity iron uptake and involved in mediating resistance to copper ion toxicity, belongs to class of integral membrane multicopper oxidases	Null mutant is viable, defective for high affinity Fe(II) uptake	S000004662
YLR342W	FKS1			**++**				CND1|CWH53|ETG1|GSC1|PBR1	Catalytic subunit of 1,3-beta-D-glucan synthase, functionally redundant with alternate catalytic subunit Gsc2p; binds to regulatory subunit Rho1p; involved in cell wall synthesis and maintenance; localizes to sites of cell wall remodeling	Null mutant is viable, growth defect, sensitive to FK506, cyclosporin A, and echinocandin	S000004334
YGR052W	FMP48				**+**	**+**			Localized to the mitochondrion, interacts with TORC1 complex	Null mutant is viable, abnormal vaculoar morphology	S000003284
YBR021W	FUR4			**+**			**+**		Uracil permease, localized to the plasma membrane; expression is tightly regulated by uracil levels and environmental cues	Null mutant is viable, abnormal bud morphology	S000000225
YMR307W	GAS1	**+**	**+**	**+**			**+**	CWH52|GGP1	Beta-1.3-glucanosyltransferase, required for cell wall assembly; localizes to the cell surface via a glycosylphosphatidylinositol (GPI) anchor	Null mutant is viable, cell wall defect, decreased growth rate, abnormal vacuolar morphology	S000004924
YCL011C	GBP2			**+**				RLF6	Poly(A+) RNA-binding protein, involved in the export of mRNAs from the nucleus to the cytoplasm; similar to Hrb1p and Npl3p; also binds single-stranded telomeric repeat sequence in vitro	Null mutant is viable, thermotolerant	S000000517
YKR026C	GCN3			**+**				AAS2	Alpha subunit of the translation initiation factor eIF2B, the guanine-nucleotide exchange factor for eIF2; activity subsequently regulated by phosphorylated eIF2; first identified as a positive regulator of GCN4 expression	Null mutant is viable, fails to derepress amino acid-regulated genes	S000001734
YGR252W	GCN5			**+**				ADA4|SWI9	Histone acetyltransferase, acetylates lysine 14 on histone H3; catalytic subunit of the ADA and SAGA histone acetyltransferase complexes; founding member of the Gcn5p-related N-acetyltransferase superfamily	Null mutant is viable, grows poorly on minimal media, sensitive to many drugs	S000003484
YNL153C	GIM3	**+**	**+**	**+**			**+**	PFD4	Subunit of the heterohexameric cochaperone prefoldin complex which binds specifically to cytosolic chaperonin and transfers target proteins to it	Null mutant is viable, cold sensitive, benomyl sensitive, increased chromsome loss rate	S000005097
YEL003W	GIM4			**+**				PFD2	Subunit of the heterohexameric cochaperone prefoldin complex which binds specifically to cytosolic chaperonin and transfers target proteins to it	Null mutant is viable, cold sensitive, benomyl sensitive, increased chromsome loss rate	S000000729
YML094W	GIM5		**+**					PFD5	Subunit of the heterohexameric cochaperone prefoldin complex which binds specifically to cytosolic chaperonin and transfers target proteins to it	Null mutant is viable, cold sensitive, benomyl sensitive, increased chromsome loss rate	S000004559
YER133W	GLC7†				**+**		**+**	CID1|DIS2|DIS2S1|PP1	Catalytic subunit of type 1 serine/threonine protein phosphatase, involved in many processes including glycogen metabolism, sporulation, and mitosis; interacts with multiple regulatory subunits; predominantly isolated with Sds22p	Null mutant is inviable, defective actin cytoskeleton and endocytosis, heat and cold sensitive, increased chromosome loss rate	S000000935
YDL035C	GPR1			**+**			**+**		Plasma membrane G protein coupled receptor (GPCR) that interacts with the heterotrimeric G protein alpha subunit, Gpa2p, and with Plc1p; sensor that integrates nutritional signals with the modulation of cell fate via PKA and cAMP synthesis	Null mutant is viable, decreased cell size, increased lifespan	S000002193
YLR258W	GSY2			**+**					Glycogen synthase, similar to Gsy1p; expression induced by glucose limitation, nitrogen starvation, heat shock, and stationary phase; activity regulated by cAMP-dependent, Snf1p and Pho85p kinases as well as by the Gac1p-Glc7p phosphatase	Null mutant is viable, gsy1 gsy2 double mutant defective in glycogen deposition	S000004248
YOR070C	GYP1			**+**				YOR29-21	Cis-golgi GTPase-activating protein (GAP) for the Rab family members Ypt1p (in vivo) and for Ypt1p, Sec4p, Ypt7p, and Ypt51p (in vitro); involved in vesicle docking and fusion	Null mutant is viable, sensitive to many drugs	S000005596
YBR009C	HHF1			**+**					One of two identical histone H4 proteins (see also HHF2); core histone required for chromatin assembly and chromosome function; contributes to telomeric silencing; N-terminal domain involved in maintaining genomic integrity	Null mutant is viable, increased chromosome loss rate, decreased telomere length	S000000213
YJR075W	HOC1	**+**	**+**	**+**			**+**		Alpha-1,6-mannosyltransferase involved in cell wall mannan biosynthesis; subunit of a Golgi-localized complex that also contains Anp1p, Mnn9p, Mnn11p, and Mnn10p; identified as a suppressor of a cell lysis sensitive pkc1-371 allele	Null mutant is viable, sensitive to many drugs	S000003836
YLR113W	HOG1			**+**	**+**	**+**	**+**	SSK3	Mitogen-activated protein kinase involved in osmoregulation via three independent osmosensors; mediates the recruitment and activation of RNA Pol II at Hot1p-dependent promoters; localization regulated by Ptp2p and Ptp3p	Null mutant is viable, unable to grow in high osmolarity media	S000004103
YPL204W	HRR25†				**+**				Protein kinase involved in regulating diverse events including vesicular trafficking, gene expression, DNA repair, and chromosome segregation; binds the CTD of RNA pol II; homolog of mammalian casein kinase 1delta (CK1delta)	Null mutant is viable, slow growth, sensitive to DNA damage	S000006125
YKL101W	HSL1				**+**			NIK1	Nim1p-related protein kinase that regulates the morphogenesis and septin checkpoints; associates with the assembled septin filament; required along with Hsl7p for bud neck recruitment, phosphorylation, and degradation of Swe1p	Null mutant is viable, abnormal bud morphology, G2/M delay	S000001584
YLL026W	HSP104			**+**					Heat shock protein that cooperates with Ydj1p (Hsp40) and Ssa1p (Hsp70) to refold and reactivate previously denatured, aggregated proteins; responsive to stresses including: heat, ethanol, and sodium arsenite; involved in [PSI+] propagation	Null mutant is viable, defective in induced thermotolerance	S000003949
YPL240C	HSP82			**+**				HSP83|HSP90	Cytoplasmic chaperone (Hsp90 family) required for pheromone signaling and negative regulation of Hsf1p; docks with the mitochondrial import receptor Tom70p for preprotein delivery; interacts with co-chaperones Cns1p, Cpr6p, Cpr7p, and Sti1p	Null mutant is viable, temperature sensitive	S000006161
YOL012C	HTZ1			**+**				H2A.F/Z|H2AZ|HTA3	Histone variant H2AZ, exchanged for histone H2A in nucleosomes by the SWR1 complex; involved in transcriptional regulation through prevention of the spread of silent heterochromatin	Null mutant is viable, loss of silencing at telomeres, sensitive to many drugs	S000005372
YGL253W	HXK2			**+**				HEX1|HKB|SCI2	Hexokinase isoenzyme 2 that catalyzes phosphorylation of glucose in the cytosol; predominant hexokinase during growth on glucose; functions in the nucleus to repress expression of HXK1 and GLK1 and to induce expression of its own gene	Null mutant is viable, fails to show glucose repression	S000003222
YHR094C	HXT1			**+**				HOR4	Low-affinity glucose transporter of the major facilitator superfamily, expression is induced by Hxk2p in the presence of glucose and repressed by Rgt1p when glucose is limiting	Null mutant is viable, decreased transport of glucose	S000001136
YOR136W	IDH2			**+**					Subunit of mitochondrial NAD(+)-dependent isocitrate dehydrogenase, which catalyzes the oxidation of isocitrate to alpha-ketoglutarate in the TCA cycle	Null mutant is viable, G1 delay, increased thermotolerance	S000005662
YLR384C	IKI3			**+**				ELP1|TOT1	Subunit of RNA polymerase II elongator histone acetyltransferase complex, involved in maintaining its structural integrity; negatively regulates exocytosis independent of transcription, homolog of human familial dysautonomia (FD) protein	Null mutant is viable, insensitive tokiller toxin, G1 delay	S000004376
YJR016C	ILV3†				**+**				Dihydroxyacid dehydratase, catalyzes third step in the common pathway leading to biosynthesis of branched-chain amino acids	Null mutant is viable, isoleucine and valine auxotroph	S000003777
YPL209C	IPL1†				**+**			PAC15	Aurora kinase involved in regulating kinetochore-microtubule attachments, associates with Sli15p, which stimulates Ipl1p kinase activity and promotes its association with the mitotic spindle, potential Cdc28p substrate	Null mutant is inviable, temperature sensitive mutant has incrwased chromosome loss rate	S000006130
YHR079C	IRE1			**++**	**+**	**+**		ERN1	Serine-threonine kinase and endoribonuclease; transmembrane protein that initiates the unfolded protein response signal by regulating synthesis of Hac1p through HAC1 mRNA splicing	Null mutant is viable, sensitive to ER stress ER	S000001121
YPR106W	ISR1				**+**				Predicted protein kinase, overexpression causes sensitivity to staurosporine, which is a potent inhibitor of protein kinase C	Null mutant is viable, exacerbates phenotype of a temperature-sensitive allele of PKC1	S000006310
YER110C	KAP123			**+**				YRB4	Karyopherin beta, mediates nuclear import of ribosomal proteins prior to assembly into ribosomes and import of histones H3 and H4; localizes to the nuclear pore, nucleus, and cytoplasm; exhibits genetic interactions with RAI1	Null mutant is viable, defective nuclear import	S000000912
YPR141C	KAR3			**+**				OSR11	Minus-end-directed microtubule motor that functions in mitosis and meiosis, localizes to the spindle pole body and localization is dependent on functional Cik1p, required for nuclear fusion during mating; potential Cdc28p substrate	Null mutant is viable, defective in nuclear fusion	S000006345
YGL173C	KEM1			**+**				DST2|RAR5|SEP1|SKI1|XRN1	Evolutionarily-conserved 5--3- exonuclease component of cytoplasmic processing (P) bodies involved in mRNA decay; plays a role in microtubule-mediated processes, filamentous growth, ribosomal RNA maturation, and telomere maintenance	Null mutant is viable, abnormal vaculoar morphology, reduced lifespan, increased chromosome loss rate	S000003141
YIL125W	KGD1			**+**				OGD1	Component of the mitochondrial alpha-ketoglutarate dehydrogenase complex, which catalyzes a key step in the tricarboxylic acid (TCA) cycle, the oxidative decarboxylation of alpha-ketoglutarate to form succinyl-CoA	Null mutant is viable, respiration deficient	S000001387
YDL108W	KIN28†				**+**		**+**		Serine/threonine protein kinase, subunit of the transcription factor TFIIH; involved in transcription initiation at RNA polymerase II promoters	Null mutant is inviable	S000002266
YNL322C	KRE1	**+**	**+**	**++**			**+**		Cell wall glycoprotein involved in beta-glucan assembly; serves as a K1 killer toxin membrane receptor	Null mutant is viable, reduced cell wall biosynthesis	S000005266
YHR082C	KSP1				**+**	**+**			Serine/threonine protein kinase; associates with TORC1; overproduction causes allele-specific suppression of the prp20-10 mutation	Null mutant is viable, increased autophagy	S000001124
YGR040W	KSS1†				**+**				Mitogen-activated protein kinase (MAPK) involved in signal transduction pathways that control filamentous growth and pheromone response	Null mutant is viable, decreased filamentous and invasive growth	S000003272
YNL071W	LAT1			**+**				ODP2|PDA2	Dihydrolipoamide acetyltransferase component (E2) of pyruvate dehydrogenase complex, which catalyzes the oxidative decarboxylation of pyruvate to acetyl-CoA	Null mutant is viable, increased rate of petite formation	S000005015
YJL134W	LCB3			**+**			**+**	LBP1|YSR2	Long-chain base-1-phosphate phosphatase, regulates ceramide and long-chain base phosphates levels, involved in incorporation of exogenous long chain bases in sphingolipids	Null mutant is viable, reduced rate of exogenous long chain base incorporation into sphingolipids	S000003670
YPL055C	LGE1	**+**	**+**	**+**			**+**		Protein of unknown function; null mutant forms abnormally large cells	Null mutant is viable, increased cell size	S000005976
YNL006W	LST8†				**+**				Protein required for the transport of amino acid permease Gap1p from the Golgi to the cell surface; component of the TOR signaling pathway; associates with both Tor1p and Tor2p; contains a WD-repeat	Null mutant is inviable, reduction-of-function mutations cause sensitivity to rapamycin	S000004951
YGL086W	MAD1			**+**					Coiled-coil protein involved in the spindle-assembly checkpoint; phosphorylated by Mps1p upon checkpoint activation which leads to inhibition of the activity of the anaphase promoting complex; forms a complex with Mad2p	Null mutant is viable, benomyl sensitive, increased chromosome loss rate	S000003054
YNL307C	MCK1			**+**	**+**			YPK1	Protein serine/threonine/tyrosine (dual-specificity) kinase involved in control of chromosome segregation and in regulating entry into meiosis; related to mammalian glycogen synthase kinases of the GSK-3 family	Null mutant is viable, benomyl sensitive, increased chromosome loss rate, thermosensitive	S000005251
YDR318W	MCM21			**+**				CTF5	Protein involved in minichromosome maintenance; component of the COMA complex (Ctf19p, Okp1p, Mcm21p, Ame1p) that bridges kinetochore subunits that are in contact with centromeric DNA and the subunits bound to microtubules	Null mutant is viable, benomyl sensitive, increased chromosome loss rate	S000002726
YKL085W	MDH1			**+**			**+**		Mitochondrial malate dehydrogenase, catalyzes interconversion of malate and oxaloacetate; involved in the tricarboxylic acid (TCA) cycle	Null mutant is viable, severe respiratory growth defect	S000001568
YBR136W	MEC1†				**+**			ESR1|SAD3	Genome integrity checkpoint protein and PI kinase superfamily member; signal transducer required for cell cycle arrest and transcriptional responses prompted by damaged or unreplicated DNA; monitors and participates in meiotic recombination	Null mutant is inviable, reduction-of-function mutant has elevated mitotic recombination and decreased telomere length	S000000340
YDL005C	MED2			**+**					Subunit of the RNA polymerase II mediator complex; associates with core polymerase subunits to form the RNA polymerase II holoenzyme; essential for transcriptional regulation	Null mutant is viable, unable to grow on galactose	S000002163
YPR167C	MET16			**+**					3--phosphoadenylsulfate reductase, reduces 3--phosphoadenylyl sulfate to adenosine-3-,5--bisphosphate and free sulfite using reduced thioredoxin as cosubstrate, involved in sulfate assimilation and methionine metabolism	Null mutant is viable, methionine auxotroph	S000006371
YOR231W	MKK1			**+**	**+**	**+**		SSP32	Mitogen-activated kinase kinase involved in protein kinase C signaling pathway that controls cell integrity; upon activation by Bck1p phosphorylates downstream target, Slt2p; functionally redundant with Mkk2p	Null mutant is viable, cannot grow on glycerol, sensitive to nitrogen starvation, temperature sensitive cell wall defect	S000005757
YLR320W	MMS22			**+**				SLM2	Protein involved in resistance to ionizing radiation; acts with Mms1p in a repair pathway that may be involved in resolving replication intermediates or preventing the damage caused by blocked replication forks	Null mutant is viable, sensitive DNA damage	S000004312
YDL028C	MPS1†				**+**			RPK1	Dual-specificity kinase required for spindle pole body (SPB) duplication and spindle checkpoint function; substrates include SPB proteins Spc42p, Spc110p, and Spc98p, mitotic exit network protein Mob1p, and checkpoint protein Mad1p	Null mutant is inviable, accumulates cells with less than 1N DNA content, required for sporulation	S000002186
YCL061C	MRC1	**+**	**+**	**+**			**+**	YCL060C	S-phase checkpoint protein found at replication forks, required for DNA replication; also required for Rad53p activation during DNA replication stress, where it forms a replication-pausing complex with Tof1p and is phosphorylated by Mec1p; protein involved in replication checkpoint	Null mutant is viable, sensitive to DNA damage, increased chromsome loss rate, anaphase delay	S000000566
YKL009W	MRT4			**+**					Protein involved in mRNA turnover and ribosome assembly, localizes to the nucleolus	Null mutant is viable, decreased mRNA decay rates	S000001492
YDR335W	MSN5			**+**				KAP142|STE21	Karyopherin involved in nuclear import and export; shown to be responsible for nuclear import of replication protein A and for export of several proteins including Swi6p, Far1p, and Pho4p; cargo dissociation involves binding to RanGTP	Null mutant is viable, defective nuclear import	S000002743
YNR049C	MSO1			**+**					Probable component of the secretory vesicle docking complex, acts at a late step in secretion; shows genetic and physical interactions with Sec1p and is enriched in microsomal membrane fractions; required for sporulation	Null mutant is viable, accumulates secretory vesicles in bud	S000005332
YDL040C	NAT1			**+**				AAA1	Subunit of the N-terminal acetyltransferase NatA (Nat1p, Ard1p, Nat5p); N-terminally acetylates many proteins, which influences multiple processes such as the cell cycle, heat-shock resistance, mating, sporulation, and telomeric silencing	Null mutant is viable, reduced acetyltransferase activity, defective mating type locus silencing, fails to enter G0	S000002198
YDR162C	NBP2			**+**			**+**		Protein involved in the HOG (high osmolarity glycerol) pathway, negatively regulates Hog1p by recruitment of phosphatase Ptc1p the Pbs2p-Hog1p complex, found in the nucleus and cytoplasm, contains an SH3 domain that binds Pbs2p	Null mutant is viable	S000002569
YML120C	NDI1			**+**					NADH:ubiquinone oxidoreductase, transfers electrons from NADH to ubiquinone in the respiratory chain but does not pump protons, in contrast to the higher eukaryotic multisubunit respiratory complex I; homolog of human AMID	Null mutant is viable, decreased apoptosis, replicative lifespan and respiratory growth	S000004589
YKL171W	NNK1				**+**	**+**			Nitrogen Network Kinase - Protein kinase	Null mutant is viable, overexpression causes hypersensitivity to rapamycin	S000001654
YNL175C	NOP13	**+**					**+**		Protein of unknown function, localizes to the nucleolus and nucleoplasm; contains an RNA recognition motif (RRM) and has similarity to Nop12p, which is required for processing of pre-18S rRNA	Null mutant is viable	S000005119
YER002W	NOP16	**+**					**+**		Constituent of 66S pre-ribosomal particles, involved in 60S ribosomal subunit biogenesis	Null mutant is viable, defect in 60S ribosomal subunit biogenesis	S000000804
YNL183C	NPR1			**+**	**+**	**+**			Protein kinase that stabilizes several plasma membrane amino acid transporters by antagonizing their ubiquitin-mediated degradation	Null mutant is viable, defect in ammonia-sensitive amino acid permeases	S000005127
YDR001C	NTH1			**+**					Neutral trehalase, degrades trehalose; required for thermotolerance and may mediate resistance to other cellular stresses; may be phosphorylated by Cdc28p	Null mutant is viable	S000002408
YDR150W	NUM1	**+**	**+**	**+**			**+**	PAC12	Protein required for nuclear migration, localizes to the mother cell cortex and the bud tip; may mediate interactions of dynein and cytoplasmic microtubules with the cell cortex	Null mutant is viable, defective nuclear segregation	S000002557
YKL068W	NUP100			**+**				NSP100	Subunit of the nuclear pore complex (NPC) that is localized to both sides of the pore; contains a repetitive GLFG motif that interacts with mRNA export factor Mex67p and with karyopherin Kap95p; homologous to Nup116p	Null mutant is viable, defective nucelar transport	S000001551
YGR078C	PAC10	**+**	**+**	**+**			**+**	GIM2|PFD3|RKS2	Part of the heteromeric co-chaperone GimC/prefoldin complex, which promotes efficient protein folding	Null mutant is viable, benomyl sensitive, cold sensitive	S000003310
YBR279W	PAF1			**+**					RNA polymerase II-associated protein, defines a large complex that is biochemically and functionally distinct from the Srb-Mediator form of Pol II holoenzyme and is required for full expression of a subset of cell cycle-regulated genes	Null mutant is viable, sensitive to many drugs	S000000483
YJL128C	PBS2				**+**			HOG4|SFS4|SSK4	MAP kinase kinase that plays a pivotal role in the osmosensing signal-transduction pathway, activated under severe osmotic stress	Null mutant is viable, sensitive to osmotic stress	S000003664
YGL248W	PDE1			**+**					Low-affinity cyclic AMP phosphodiesterase, controls glucose and intracellular acidification-induced cAMP signaling, target of the cAMP-protein kinase A (PKA) pathway; glucose induces transcription and inhibits translation	Null mutant is viable, accumulates cAMP, sensitive to rapamycin	S000003217
YMR231W	PEP5			**+**				END1|VAM1|VPL9|VPS11|VPT11	Peripheral vacuolar membrane protein required for protein trafficking and vacuole biogenesis; forms complex with Pep3p that promotes vesicular docking/fusion reactions in conjunction with SNARE proteins, also interacts with Pep7p	Null mutant is viable, abnormal vaculoar morphology	S000004844
YOL147C	PEX11			**+**				PMP24|PMP27	Peroxisomal membrane protein required for peroxisome proliferation and medium-chain fatty acid oxidation, most abundant protein in the peroxisomal membrane, regulated by Adr1p and Pip2p-Oaf1p, promoter contains ORE and UAS1-like elements	Null mutant is viable, abnormal peroxisomal morphology	S000005507
YMR205C	PFK2			**+**	**+**				Beta subunit of heterooctameric phosphofructokinase involved in glycolysis, indispensable for anaerobic growth, activated by fructose-2,6-bisphosphate and AMP, mutation inhibits glucose induction of cell cycle-related genes	Null mutant is viable, slow growth, decreased glucose utilization	S000004818
YOL136C	PFK27				**+**			PFK-2	6-phosphofructo-2-kinase, has negligible fructose-2,6-bisphosphatase activity, inhibited by phosphoenolpyruvate and sn-glycerol 3-phosphate, expression induced by glucose and sucrose, transcriptional regulation involves protein kinase A	Null mutant is viable, abnormal vacuolar morphology, resistant to killer toxin	S000005496
YPL031C	PHO85†				**+**			LDB15	Cyclin-dependent kinase, with ten cyclin partners; involved in environmental stress response; in phosphate-rich conditions, Pho85p-Pho80p complex phosphorylates Pho4p which in turn represses PHO5	Null mutant is viable, abnormal morphology, increased chromosome loss rate, increased lifespan, sensitivity to many drugs	S000005952
YBL105C	PKC1†				**+**		**+**	CLY15|HPO2|STT1	Protein serine/threonine kinase essential for cell wall remodeling during growth; localized to sites of polarized growth and the mother-daughter bud neck; homolog of the alpha, beta, and gamma isoforms of mammalian protein kinase C (PKC)	Null mutant is inviable, arrests growth with small buds due to cell wall lysis	S000000201
YGL167C	PMR1			**+**				BSD1|LDB1	High affinity Ca2+/Mn2+ P-type ATPase required for Ca2+ and Mn2+ transport into Golgi; involved in Ca2+ dependent protein sorting and processing; mutations in human homolog ATP2C1 cause acantholytic skin condition Hailey-Hailey disease	Null mutant is viable, inositol auxotroph, abnormal vacuolar morphology, decreased lifespan	S000003135
YJR043C	POL32		**+**	**+**					Third subunit of DNA polymerase delta, involved in chromosomal DNA replication; required for error-prone DNA synthesis in the presence of DNA damage and processivity; interacts with Hys2p, PCNA (Pol30p), and Pol1p	Null mutant is viable, increased chromosome loss rate, cold sensitive, DNA damage sensitive	S000003804
YMR129W	POM152			**+**					Nuclear pore membrane glycoprotein; may be involved in duplication of nuclear pores and nuclear pore complexes during S-phase;	Null mutant is viable, defective nuclear transport	S000004736
YNR052C	POP2			**+**				CAF1	RNase of the DEDD superfamily, subunit of the Ccr4-Not complex that mediates 3- to 5- mRNA deadenylation	Null mutant is viable, increased chromosome loss rate, sensitive to many drugs	S000005335
YDL134C	PPH21		**+**	**+**			**+**	PPH1	Catalytic subunit of protein phosphatase 2A, functionally redundant with Pph22p; methylated at C terminus; forms alternate complexes with several regulatory subunits; involved in signal transduction and regulation of mitosis	Null mutant is viable, increased chromosome loss rate, abnormal lipid particles	S000002292
YML016C	PPZ1			**+**			**+**		Serine/threonine protein phosphatase Z, isoform of Ppz2p; involved in regulation of potassium transport, which affects osmotic stability, cell cycle progression, and halotolerance	Null mutant is viable, increased cell size, resistant to ionic stress	S000004478
YGR135W	PRE9			**+**					20S proteasome beta-type subunit; the only nonessential 20S subunit	Null mutant is viable	S000003367
YDL214C	PRR2				**+**				Protein kinase with a possible role in MAP kinase signaling in the pheromone response pathway	Null mutant is viable, increased filamentous growth, decreased pheromone response	S000002373
YJL166W	QCR8			**+**				COR5	Subunit 8 of ubiquinol cytochrome-c reductase complex, which is a component of the mitochondrial inner membrane electron transport chain; oriented facing the intermembrane space; expression is regulated by Abf1p and Cpf1p	Null mutant is viable, respiration deficient	S000003702
YEL037C	RAD23			**+**					Protein with ubiquitin-like N terminus, recognizes and binds damaged DNA (with Rad4p) during nucleotide excision repair; regulates Rad4p levels, subunit of Nuclear Excision Repair Factor 2 (NEF2); homolog of human HR23A and HR23B proteins	Null mutant is viable, increased chromosome loss rate, defective DNA damage response	S000000763
YNL250W	RAD50			**+**					Subunit of MRX complex, with Mre11p and Xrs2p, involved in processing double-strand DNA breaks in vegetative cells, initiation of meiotic DSBs, telomere maintenance, and nonhomologous end joining	Null mutant is viable, increased chromosome loss rate, defective DNA damage response	S000005194
YML032C	RAD52			**+**					Protein that stimulates strand exchange by facilitating Rad51p binding to single-stranded DNA; anneals complementary single-stranded DNA; involved in the repair of double-strand breaks in DNA during vegetative growth and meiosis	Null mutant is viable, deficient in homologous recombination	S000004494
YPL153C	RAD53†				**+**		**+**	LSD1|MEC2|SPK1	Protein kinase, required for cell-cycle arrest in response to DNA damage; activated by trans autophosphorylation when interacting with hyperphosphorylated Rad9p	Null mutant is inviable, point mutants sensitive to DNA-damaging agents	S000006074
YOR265W	RBL2			**+**					Protein involved in microtubule morphogenesis, required for protection from excess free beta-tubulin; proposed to be involved the folding of beta-tubulin	Null mutant is viable, sensitive to many stresses	S000005791
YDR195W	REF2			**+**					RNA-binding protein involved in the cleavage step of mRNA 3--end formation prior to polyadenylation, and in snoRNA maturation; part of holo-CPF subcomplex APT, which associates with 3--ends of snoRNA- and mRNA-encoding genes	Null mutant is viable, sensitive to many stresses	S000002603
YDR137W	RGP1			**+**					Subunit of a Golgi membrane exchange factor (Ric1p-Rgp1p) that catalyzes nucleotide exchange on Ypt6p	Null mutant is viable, sensitive to many stresses	S000002544
YLR039C	RIC1		**+**	**+**			**+**		Protein involved in retrograde transport to the cis-Golgi network; forms heterodimer with Rgp1p that acts as a GTP exchange factor for Ypt6p; involved in transcription of rRNA and ribosomal protein genes	Null mutant is viable, sensitive to many stresses	S000004029
YOR119C	RIO1†				**+**	**+**		RRP10	Essential serine kinase involved in cell cycle progression and processing of the 20S pre-rRNA into mature 18S rRNA	Null mutant is inviable, defective in ribosome biogenesis	S000005645
YNL207W	RIO2†				**+**	**+**			Essential serine kinase involved in the processing of the 20S pre-rRNA into mature 18S rRNA; has similarity to Rio1p	Null mutant is inviable, defective in ribosome biogenesis	S000005151
YIL066C	RNR3			**+**			**+**	DIN1|RIR3	Ribonucleotide-diphosphate reductase (RNR), large subunit; the RNR complex catalyzes the rate-limiting step in dNTP synthesis and is regulated by DNA replication and DNA damage checkpoint pathways via localization of the small subunits	Null mutant is viable, sensitive to dNTP pool depletion	S000001328
YLR371W	ROM2			**+**					GDP/GTP exchange protein (GEP) for Rho1p and Rho2p; mutations are synthetically lethal with mutations in rom1, which also encodes a GEP	Null mutant is viable, temperature and cold sensitive, increased cell size, abnormal bud morphology, sensitive to benomyl	S000004363
YBR229C	ROT2		**+**	**+**			**+**	GLS2	Glucosidase II catalytic subunit required for normal cell wall synthesis; mutations in rot2 suppress tor2 mutations, and are synthetically lethal with rot1 mutations	Null mutant is viable, abnormal vacuollar morphology, rot 1 rot2 double mutant is inviable	S000000433
YNL248C	RPA49			**+**				A49	RNA polymerase I subunit A49	Null mutant is viable, temperature and cold sensitive, abnormal nuclear morphology	S000005192
YNL067W	RPL9B			**+**			**+**		Protein component of the large (60S) ribosomal subunit, nearly identical to Rpl9Ap and has similarity to E. coli L6 and rat L9 ribosomal proteins	Null mutant is viable	S000005011
YHR027C	RPN1†			**+**				HRD2|NAS1	Non-ATPase base subunit of the 19S regulatory particle of the 26S proteasome; may participate in the recognition of several ligands of the proteasome; contains a leucine-rich repeat (LRR) domain, a site for protein?protein interactions	Null mutant is inviable; point mutants are sensitive to canavanine, global accumulation of ubiquitin-conjugates	S000001069
YHR200W	RPN10			**+**				MCB1|SUN1	Non-ATPase base subunit of the 19S regulatory particle (RP) of the 26S proteasome; N-terminus plays a role in maintaining the structural integrity of the RP; binds selectively to polyubiquitin chains; homolog of the mammalian S5a protein	Null mutant is viable, sensitive to amino acid analogs, increased ubiquitin conjugates	S000001243
YOR001W	RRP6			**+**					Exonuclease component of the nuclear exosome; contributes to the quality-control system that retains and degrades aberrant mRNAs in the nucleus	Null mutant is viable, accumulates incompletely processed mRNA, rRNA, snRNA, snoRNA species	S000005527
YLR221C	RSA3			**+**					Protein with a likely role in ribosomal maturation, required for accumulation of wild-type levels of large (60S) ribosomal subunits; binds to the helicase Dbp6p in pre-60S ribosomal particles in the nucleolus	Null mutant is viable, required for 60S ribosomal subunit biogenesis	S000004211
YLR357W	RSC2			**+**					One of 15 subunits of the -Remodel the Structure of Chromatin- (RSC) complex; required for expression of mid-late sporulation-specific genes; involved in telomere maintenance	Null mutant is viable, increased chromosome loss rate, sensitive to many drugs	S000004349
YDR388W	RVS167			**+**					Actin-associated protein, subunit of a complex (Rvs161p-Rvs167p) involved in regulation of actin cytoskeleton, endocytosis, and viability following starvation or osmotic stress; homolog of mammalian amphiphysin	Null mutant is viable, reduced viability upon starvation	S000002796
YDR159W	SAC3			**+**				LEP1	Nuclear pore-associated protein, forms a complex with Thp1p that is involved in transcription and in mRNA export from the nucleus	Null mutant is viable, increased cell size, sensitive to many drugs, cold and temperature sensitive	S000002566
YDR502C	SAM2			**+**				ETH2	S-adenosylmethionine synthetase, catalyzes transfer of the adenosyl group of ATP to the sulfur atom of methionine; one of two differentially regulated isozymes (Sam1p and Sam2p)	Null mutant is viable, sam1 sam2 double mutant is AdoMet auxotroph	S000002910
YMR263W	SAP30	**+**	**+**	**+**			**+**		Subunit of a histone deacetylase complex, along with Rpd3p and Sin3p, that is involved in silencing at telomeres, rDNA, and silent mating-type loci; involved in telomere maintenance	Null mutant is viable, decreased mating efficiency and telomere length	S000004876
YOR329C	SCD5†				**+**			FTB1	Protein required for normal cortical actin organization and endocytosis; multicopy suppressor of clathrin deficiency; acts as a targeting subunit for protein phosphatase type 1	Null mutant is inviable, point mutant has abnormal morphology and decreased endocytosis	S000005856
YHR205W	SCH9†				**+**		**+**	KOM1	Protein kinase that regulates signal transduction activity and G1 progression, controls cAPK activity, required for nitrogen activation of the FGM pathway, involved in life span regulation, homologous to mammalian Akt/PKB	Null mutant is viable, grows slowly, increased lifespan	S000001248
YLL041C	SDH2			**+**				ACN17	Iron-sulfur protein subunit of succinate dehydrogenase (Sdh1p, Sdh2p, Sdh3p, Sdh4p), which couples the oxidation of succinate to the transfer of electrons to ubiquinone	Null mutant is viable, fails to grow on nonfermentable carbon sources, increased lifespan	S000003964
YKL193C	SDS22†				**+**			EGP1	Conserved nuclear regulatory subunit of Glc7p type 1 protein serine-threonine phosphatase (PP1), functions positively with Glc7p to promote dephosphorylation of nuclear substrates required for chromosome transmission during mitosis	Null mutant is inviable, frameshift mutation causes heat and cold sensitivity, and increased chromosome loss rate	S000001676
YLR268W	SEC22			**+**				SLY2|TSL26	R-SNARE protein; assembles into SNARE complex with Bet1p, Bos1p and Sed5p; cycles between the ER and Golgi complex; involved in anterograde and retrograde transport between the ER and Golgi; synaptobrevin homolog	Null mutant is viable, cold and heat sensitive, abnormal ER morphology, cell wall defects	S000004258
YBR171W	SEC66			**+**				HSS1|SEC71	Non-essential subunit of Sec63 complex (Sec63p, Sec62p, Sec66p and Sec72p); with Sec61 complex, Kar2p/BiP and Lhs1p forms a channel competent for SRP-dependent and post-translational SRP-independent protein targeting and import into the ER	Null mutant is viable, defective in filamentous growth, sensistive to many drugs	S000000375
*pdr1Δpdr3Δ*	Sensitized strain	**+**	**+**	**+**	**+**	**+**					
YOR184W	SER1			**+**				ADE9	3-phosphoserine aminotransferase, catalyzes the formation of phosphoserine from 3-phosphohydroxypyruvate, required for serine and glycine biosynthesis; regulated by the general control of amino acid biosynthesis mediated by Gcn4p	Null mutant is viable, serine auxotroph	S000005710
YJL168C	SET2			**+**				EZL1	Histone methyltransferase with a role in transcriptional elongation, methylates a lysine residue of histone H3; associates with the C-terminal domain of Rpo21p; histone methylation activity is regulated by phosphorylation status of Rpo21p	Null mutant is viable, lacks methylation of histone H3 at Lys36	S000003704
YLR403W	SFP1†				**+**				Transcription factor that controls expression of many ribosome biogenesis genes in response to nutrients and stress, regulates G2/M transitions during mitotic cell cycle and DNA-damage response, involved in cell size modulation	Null mutant is viable, grows slowly, defective in ribosome biogenesis gene expression	S000004395
YLR058C	SHM2		**+**	**+**				SHMT2	Cytosolic serine hydroxymethyltransferase, involved in one-carbon metabolism	Null mutant is viable, shm2 srp40 ade3 triple mutnat is inviable	S000004048
YBL061C	SKT5			**+**				CAL2|CHS4|CSD4	Activator of Chs3p (chitin synthase III), recruits Chs3p to the bud neck via interaction with Bni4p; has similarity to Shc1p, which activates Chs3p during sporulation	Null mutant is viable, reduced cell wall chitin deposition	S000000157
YIL147C	SLN1†				**+**		**+**	YPD2	Histidine kinase osmosensor that regulates a MAP kinase cascade; transmembrane protein with an intracellular kinase domain that signals to Ypd1p and Ssk1p, thereby forming a phosphorelay system similar to bacterial two-component regulators	Null mutant is inviable, constitutive activation of the HOG1 MAPK cascade	S000001409
YHR030C	SLT2		**+**	**++**	**+**	**+**	**+**	BYC2|MPK1|SLK2	Serine/threonine MAP kinase involved in regulating the maintenance of cell wall integrity and progression through the cell cycle; regulated by the PKC1-mediated signaling pathway	Null mutant is viable, temperature sensitive cell lysis defect	S000001072
YGR229C	SMI1	**+**	**+**	**+**			**+**	KNR4	Protein involved in the regulation of cell wall synthesis; proposed to be involved in coordinating cell cycle progression with cell wall integrity	Null mutant is viable, sensitive to osmotic stress, sensitive to many chemicals	S000003461
YAL030W	SNC1			**+**					Vesicle membrane receptor protein (v-SNARE) involved in the fusion between Golgi-derived secretory vesicles with the plasma membrane; proposed to be involved in endocytosis; member of the synaptobrevin/VAMP family of R-type v-SNARE proteins	Null mutant is viable; snc1 snc2 double mutant is deficient in bulk secretion	S000000028
YDR477W	SNF1†				**+**			CAT1|CCR1|GLC2|HAF3|PAS14	AMP-activated serine/threonine protein kinase found in a complex containing Snf4p and members of the Sip1p/Sip2p/Gal83p family; required for transcription of glucose-repressed genes, thermotolerance, sporulation, and peroxisome biogenesis	Null mutant is viable, fails to accumulate glycogen; sucrose nonfermenting	S000002885
YOR290C	SNF2			**+**				GAM1|HAF1|SWI2|TYE3	Catalytic subunit of the SWI/SNF chromatin remodeling complex involved in transcriptional regulation; contains DNA-stimulated ATPase activity; functions interdependently in transcriptional activation with Snf5p and Snf6p	Null mutant is viable, defects in chromatin remodeling and transcriptional regulation, inability to switch mating type, increased sensitivity to DNA damage, defective cell wall	S000005816
YIL073C	SPO22			**+**			**+**		Meiosis-specific protein with similarity to phospholipase A2, involved in completion of nuclear divisions during meiosis; induced early in meiosis	Null mutant is viable, defective spore production	S000001335
YDR392W	SPT3			**+**					Subunit of the SAGA and SAGA-like transcriptional regulatory complexes, interacts with Spt15p to activate transcription of some RNA polymerase II-dependent genes, also functions to inhibit transcription at some promoters	Null mutant is viable, defective in mating and sporulation, Ty transcription	S000002800
YNL224C	SQS1	**+**					**+**		SQuelch of Splicing suppression	Null mutant is viable, facilitates 18S rRNA maturation	S000005168
YKR091W	SRL3			**+**					Cytoplasmic protein that, when overexpressed, suppresses the lethality of a rad53 null mutation; potential Cdc28p substrate	Null mutant is viable	S000001799
YJL092W	SRS2			**+**				RADH|RADH1|HPR5	DNA helicase and DNA-dependent ATPase involved in DNA repair, required for proper timing of commitment to meiotic recombination and the transition from Meiosis I to Meiosis II; potential Cdc28p substrate	Null mutant is viable, causes RAD52-dependent hyperrecombination	S000003628
YNL209W	SSB2			**+**				YG103	Cytoplasmic ATPase that is a ribosome-associated molecular chaperone, functions with J-protein partner Zuo1p; may be involved in the folding of newly-synthesized polypeptide chains; member of the HSP70 family; homolog of SSB1	Null mutant is viable, ssb1 ssb2 double mutant has growth defect, hypersensitivity to protein synthesis inhibitors	S000005153
YPL106C	SSE1			**+**				LPG3|MSI3	ATPase that is a component of the heat shock protein Hsp90 chaperone complex; binds unfolded proteins; member of the heat shock protein 70 (HSP70) family; localized to the cytoplasm	Null mutant is viable, slow growth, decreased glucose utilization	S000006027
YNL222W	SSU72†				**+**				Transcription/RNA-processing factor that associates with TFIIB and cleavage/polyadenylation factor Pta1p; exhibits phosphatase activity on serine-5 of the RNA polymerase II C-terminal domain; affects start site selection in vivo	Null mutant is inviable, increased chromosome loss rate	S000005166
YMR125W	STO1			**+**				CBC1|CBP80|GCR3|SUT1	Large subunit of the nuclear mRNA cap-binding protein complex, interacts with Npl3p to carry nuclear poly(A)+ mRNA to cytoplasm; also involved in nuclear mRNA degradation and telomere maintenance; orthologous to mammalian CBP80	Null mutant is viable, defective growth on fermentable carbon sources	S000004732
YDR297W	SUR2			**+**				SYR2	Sphinganine C4-hydroxylase, catalyses the conversion of sphinganine to phytosphingosine in sphingolipid biosyntheis	Null mutant is viable, altered sphingolipid levels	S000002705
YBR231C	SWC5			**+**				AOR1	Protein of unknown function, component of the Swr1p complex that incorporates Htz1p into chromatin	Null mutant is viable, increased lifespan, abnormal bud and vacuolar morphology	S000000435
YJL187C	SWE1		**+**	**++**				WEE1	Protein kinase that regulates the G2/M transition by inhibition of Cdc28p kinase activity; localizes to the nucleus and to the daughter side of the mother-bud neck; homolog of S. pombe Wee1p; potential Cdc28p substrate	Null mutant is viable, defective for morphogenesis checkpoint	S000003723
YER111C	SWI4			**+**				ART1	DNA binding component of the SBF complex (Swi4p-Swi6p), a transcriptional activator that in concert with MBF (Mbp1-Swi6p) regulates late G1-specific transcription of targets including cyclins and genes required for DNA synthesis and repair	Null mutant is viable, defect in G1/S transcription causes G1/S delay	S000000913
YDR334W	SWR1			**+**					Swi2/Snf2-related ATPase, component of the SWR1 complex; required for the incorporation of Htz1p into chromatin	Null mutant is viable, increased lifespan and thermotolerance	S000002742
YNL128W	TEP1	**+**	**+**	**+**			**+**		Homolog of human tumor suppressor gene PTEN/MMAC1/TEP1 that has lipid phosphatase activity and is linked to the phosphatidylinositol signaling pathway; plays a role in normal sporulation	Null mutant is viable, abnormal cell wall morphology	S000005072
YDL185W	TFP1			**+**				CLS8|VMA1	Vacuolar ATPase V1 domain subunit A containing the catalytic nucleotide binding sites; protein precursor undergoes self-catalyzed splicing to yield the extein Tfp1p and the intein Vde (PI-SceI), which is a site-specific endonuclease	Null mutant is viable, abnormal vacuolar and mitochondrial morphology, reduced lipid droplets	S000002344
YPR163C	TIF3			**+**				RBL3|STM1	Translation initiation factor eIF-4B, has RNA annealing activity; contains an RNA recognition motif and binds to single-stranded RNA	Null mutant is viable, abnormal bud morphology, increased cell size	S000006367
YOL006C	TOP1		**+**	**+**			**+**	MAK1|MAK17	Topoisomerase I, nuclear enzyme that relieves torsional strain in DNA by cleaving and re-sealing the phosphodiester backbone; relaxes both positively and negatively supercoiled DNA; functions in replication, transcription, and recombination	Null mutant is viable, increased chromosome loss rate	S000005366
YJR066W	TOR1			**+**	**+**	**+**		DRR1	PIK-related protein kinase and rapamycin target; subunit of TORC1, a complex that controls growth in response to nutrients by regulating translation, transcription, ribosome biogenesis, nutrient transport and autophagy; involved in meiosis	Null mutant is viable, grows slowly, increased lifespan, thermotolerance and oxidative stress resistance, increased sensitivity to rapamycin, tor1 tor2 double mutant is inviable	S000003827
YKL203C	TOR2†				**+**	**+**		DRR2	PIK-related protein kinase and rapamycin target; subunit of TORC1, a complex that regulates growth in response to nutrients and TORC2, a complex that regulates cell-cycle dependent polarization of the actin cytoskeleton; involved in meiosis	Null mutant is inviable, defective actin cytoskeleton and endocytosis, heat and cold sensitive, increased chromosome loss rate	S000001686
YJL164C	TPK1			**+**				PKA1|SRA3	Subunit of cytoplasmic cAMP-dependent protein kinase, which contains redundant catalytic subunits Tpk1p, Tpk2p, and Tpk3p and regulatory subunit Bcy1p; promotes vegetative growth in response to nutrients; inhibits filamentous growth	Null mutant is viable, sensitive to desiccation, multicopy suppressor of ras mutant	S000003700
YJL129C	TRK1			**+**					Component of the Trk1p-Trk2p potassium transport system; 180 kDa high affinity potassium transporter	Null mutant is viable, requires added potassium	S000003665
YML124C	TUB3			**+**					Alpha-tubulin; associates with beta-tubulin (Tub2p) to form tubulin dimer, which polymerizes to form microtubules; expressed at lower level than Tub1p	Null mutant is viable, sensitive to benomyl, poor spore viability	S000004593
YFR010W	UBP6			**+**					Ubiquitin-specific protease situated in the base subcomplex of the 26S proteasome, releases free ubiquitin from branched polyubiquitin chains; deletion causes hypersensitivity to cycloheximide and other toxic compounds	Null mutant is viable, decreased lifespan, increased [Psi+] prion formation	S000001906
YMR223W	UBP8			**+**					Ubiquitin-specific protease that is a component of the SAGA (Spt-Ada-Gcn5-Acetyltransferase) acetylation complex; required for SAGA-mediated deubiquitination of histone H2B	Null mutant is viable, increased lifespan, sensitive to DNA damage and desiccation, defective in sporulation	S000004836
YOR106W	VAM3			**+**				PTH1	Syntaxin-related protein required for vacuolar assembly; functions with Vam7p in vacuolar protein trafficking; member of the syntaxin family of proteins	Null mutant is viable, defective processing of vacuolar hydrolases	S000005632
YCL069W	VBA3			**+**					Permease of basic amino acids in the vacuolar membrane	Null mutant is viable, abnormal vacuolar morphology	S000000574
YGL227W	VID30			**+**				GID1	Protein involved in proteasome-dependent catabolite degradation of fructose-1,6-bisphosphatase (FBPase); shifts the balance of nitrogen metabolism toward the production of glutamate; localized to the nucleus and the cytoplasm	Null mutant is viable, vacuolar degradation of cytosolic proteins, sensitive to starvation	S000003196
YOR270C	VPH1			**+**					Subunit of vacuolar-ATPase V0 domain, one of two isoforms (Vph1p and Stv1p); Vph1p is located in V-ATPase complexes of the vacuole while Stv1p is located in V-ATPase complexes of the Golgi and endosomes	Null mutant is viable, deficient in acidification of the vacuole	S000005796
YKR001C	VPS1			**+**				GRD1|LAM1|SPO15|VPL1|VPT26	GTPase required for vacuolar protein sorting, functions in actin cytoskeleton organization via its interaction with Sla1p; required for late Golgi-retention of some proteins including Kex2p; involved in regulating peroxisome biogenesis	Null mutant is viable, sporulation defective, abnormal organization of intracellular membranes	S000001709
YJL154C	VPS35			**+**				GRD9|VPT7	Endosomal protein that is a subunit of the membrane-associated retromer complex essential for endosome-to-Golgi retrograde transport; forms a subcomplex with Vps26p and Vps29p that selects cargo proteins for endosome-to-Golgi retrieval	Null mutant is viable, defects in vacuolar sorting	S000003690
YOR069W	VPS5			**+**				GRD2|PEP10|VPT5|YOR29-20	Nexin-1 homolog required for localizing membrane proteins from a prevacuolar/late endosomal compartment back to the late Golgi apparatus; structural component of the retromer membrane coat complex; forms a retromer subcomplex with Vps17p	Null mutant is viable, defects in vacuolar sorting	S000005595
YNL197C	WHI3	**+**					**+**		RNA binding protein that binds to and sequesters the G1 cyclin CLN3 mRNA; regulates cell fate and dose-dependently inhibits passage through Start by regulating the critical cell size requirement necessary for cell cycle progression	Null mutant is viable, defective in filamentous growth	S000005141
YOR083W	WHI5	**+**		**+**			**+**		Protein that regulates the critical cell size required for passage through Start and commitment to cell division; may act upstream of SCB binding factor (SBF) and MCB binding factor (MBF); periodically expressed in G1	Null mutant is viable, reduced cell size	S000005609
BY4741	Wild type	**+**	**+**	**+**	**+**	**+**	**+**				
YER123W	YCK3				**+**		**+**	CKI3	Palmitoylated, vacuolar membrane-localized casein kinase I isoform; negatively regulates vacuole fusion during hypertonic stress via phosphorylation of the HOPS complex subunit, Vps41p; shares overlapping essential functions with Hrr25p	Null mutant is viable; abnormal vacuolar and endomembrane system morphology	S000000925
YHR087W	YHR087W		**+**					HGI1|RTC3	Protein of unknown function involved in RNA metabolism; has structural similarity to SBDS, the human protein mutated in Shwachman-Diamond Syndrome (the yeast SBDS ortholog = SDO1); null mutation suppresses cdc13-1 temperature sensitivity; restriction of telomere capping	Null mutation is viable	S000001129
YKL067W	YNK1				**+**			NDK1	Nucleoside diphosphate kinase, catalyzes the transfer of gamma phosphates from nucleoside triphosphates, usually ATP, to nucleoside diphosphates by a mechanism that involves formation of an autophosphorylated enzyme intermediate	Null mutation is viable	S000001550
YFR003C	YPI1†				**+**				Inhibitor of the type I protein phosphatase Glc7p, which is involved in regulation of a variety of metabolic processes; overproduction causes decreased cellular content of glycogen	Null mutation is inviable, G2/M phase defect	S000001899
YLR262C	YPT6		**+**	**++**			**+**		GTPase, Ras-like GTP binding protein involved in the secretory pathway, required for fusion of endosome-derived vesicles with the late Golgi, maturation of the vacuolar carboxypeptidase Y; has similarity to the human GTPase, Rab6	Null mutant is viable, increased lifespan, abnormal vacuolar morphology, impaired utilization of nitrogen sources, impaired endocytosis	S000004252
Total Strains Screened		22	34	175	71	16	58				

*taken from Saccharomyces Genome Database.

^†^screen performed on heterozygous diploid deletion strain.

**Table 2:** Library screen strains and hits that comprise the chemical genetic matrix

**Library**	**Compounds**	**Unique cryptagen hits**	**Sentinel strains**	**Description**
Spectrum03	2000	394	89	Microsource Spectrum Collections consisting of 60% drug-like compounds, 25% natural products and 15% other bioactive molecules
Spectrum05	2000	54	87	
Spectrum08	2000	31	7	
LOPAC	1267	67	20	Library Of Pharmalogically Active Compounds
Maybridge	1000	254	32	Maybridge Hitskit 1000 selection of synthetic compounds representative of Maybridge Hitfinder library
Bioactive 1	678	421	69	Selected from Maybridge Hitfinder 50,000 compound library for growth inhibtory activity in yeast
Bioactive 2	892	213	14	Selected from Maybridge Hitfinder 50,000 compound library for growth inhibtory activity in yeast
Total	5518	1434	242	
